# Molecular insights into de novo small-molecule recognition by an intron RNA structure

**DOI:** 10.1073/pnas.2502425122

**Published:** 2025-05-08

**Authors:** Tianshuo Liu, Ling Xu, Kevin Chung, Luke J. Sisto, Jimin Hwang, Chengxin Zhang, Michael C. Van Zandt, Anna Marie Pyle

**Affiliations:** ^a^Department of Molecular, Cellular and Developmental Biology, Yale University, New Haven, CT 06511; ^b^HHMI, Chevy Chase, MD 20815; ^c^Department of Molecular Biophysics and Biochemistry, Yale University, New Haven, CT 06511; ^d^New England Discovery Partners, Branford, CT 06405; ^e^Department of Chemistry, Yale University, New Haven, CT 06511

**Keywords:** RNA-targeting ligands, high-throughput screening, cryoEM, splicing inhibitor, RNA–ligand recognition

## Abstract

Despite the promise of vastly expanding the druggable genome, rational design of RNA-targeting ligands remains challenging as it requires the rapid identification of hits and visualization of the resulting cocomplexes for guiding optimization. Here, we leveraged high-throughput screening, medicinal chemistry, and structural biology to identify a de novo splicing inhibitor against a large and highly folded fungal group I intron. High-resolution cryoEM structures of the intron in different liganded states not only reveal molecular interactions that rationalize experimental structure–activity relationship but also shed light on a unique strategy whereby RNA-associated metal ions and RNA conformation exhibit exceptional plasticity in response to small-molecule binding. This study reveals general principles that govern RNA–ligand recognition, the interplay between chemical bonding specificity, and dynamic responses within an RNA target.

## Significance

The ability to target and regulate RNA function with small molecules has vast potential, but molecular understanding of the metrics governing RNA–drug interactions is limited. Here, we present a unified study in which de novo small-molecule targeting of a large catalytic RNA was combined with high-resolution structural analysis of the optimized RNA–ligand complex, resulting in a fully structurally rationalized SAR that provides insights into strategies for molecular recognition by RNA. The optimized ligand compounds are of particular interest because they bind and inhibit an unusual type of group I intron that is important for the metabolism of pathogenic fungi, underscoring the great potential of RNA targeting in the development of pathogen-specific antimicrobials.

Targeting RNA with small molecules is an appealing drug development strategy as it promises to significantly expand the druggable genome, making possible pharmacological targeting of genes that are either noncoding or difficult to target on the protein level (1–4). Despite great interest in the RNA-targeting field and its increasing momentum, molecular discovery of functionally active RNA binders usually relies on serendipity (5, 6), and it is challenging to adapt existing target-centric approaches due to the low hit rates obtained from routinely examined chemical space (7). These issues are compounded by a lack of robust high-throughput screening (HTS)-compatible assays that monitor the biochemical or phenotypical outcomes of small-molecule binding (8). Recently, there is growing appreciation for a privileged RNA-targeting chemical space that follows the rules of drug-likeness (7, 9–11), and preliminary success in identifying functional RNA binders has been achieved (12, 13). However, assay technologies beyond affinity selection are currently limited, highlighting the need for strategies to find novel hits and thereby achieve broader success during screening campaigns.

The lack of generalizable structural methods to visualize the atomic details of RNA–ligand interactions has further restrained the rational development of RNA-targeted small molecules. The large size of functionally relevant RNA molecules and RNA complexes limits the utility of X-ray crystallography, and therefore only small RNA molecules and isolated domains tend to be amenable to this approach (5, 14–19). Additional structural methods such as small-angle X-ray scattering (SAXS) and NMR spectroscopy are valuable but constrained by low resolution and size limits, respectively (12, 20, 21). In recent years, cryogenic electron microscopy (cryoEM) has become a state-of-the-art method in deciphering small-molecule binding to large protein complexes, such as ion channel modulators (22), GPCR modulators (23), and molecular glues for targeted protein degradation (24), which has significantly advanced our understanding of molecular pharmacology. Despite the successes of resolving RNA-only structures to high resolution (25–27), determining the cryoEM structures of RNA small-molecule complexes is still in its infancy, with moderate resolution achieved so far, yielding limited molecular insights (28–30). For de novo ligands that are identified from HTS campaigns, high-resolution insights into their binding modes are urgently needed to guide subsequent rational optimization.

To unlock the vast potential of RNA targeting, we selected an unusual, highly structured group I intron that resides in the mitochondria of pathogenic fungi, and we established a systematic approach for identifying potent RNA ligands through screening and medicinal chemistry. We then employed cryoEM to obtain high-resolution structures of this structurally unique intron class in different liganded states, thereby revealing the molecular recognition strategies and dynamical consequences of de novo ligand binding to this RNA target.

## Results

### Identification of De Novo Intron Splicing Inhibition via HTS.

**Fig. 1.**
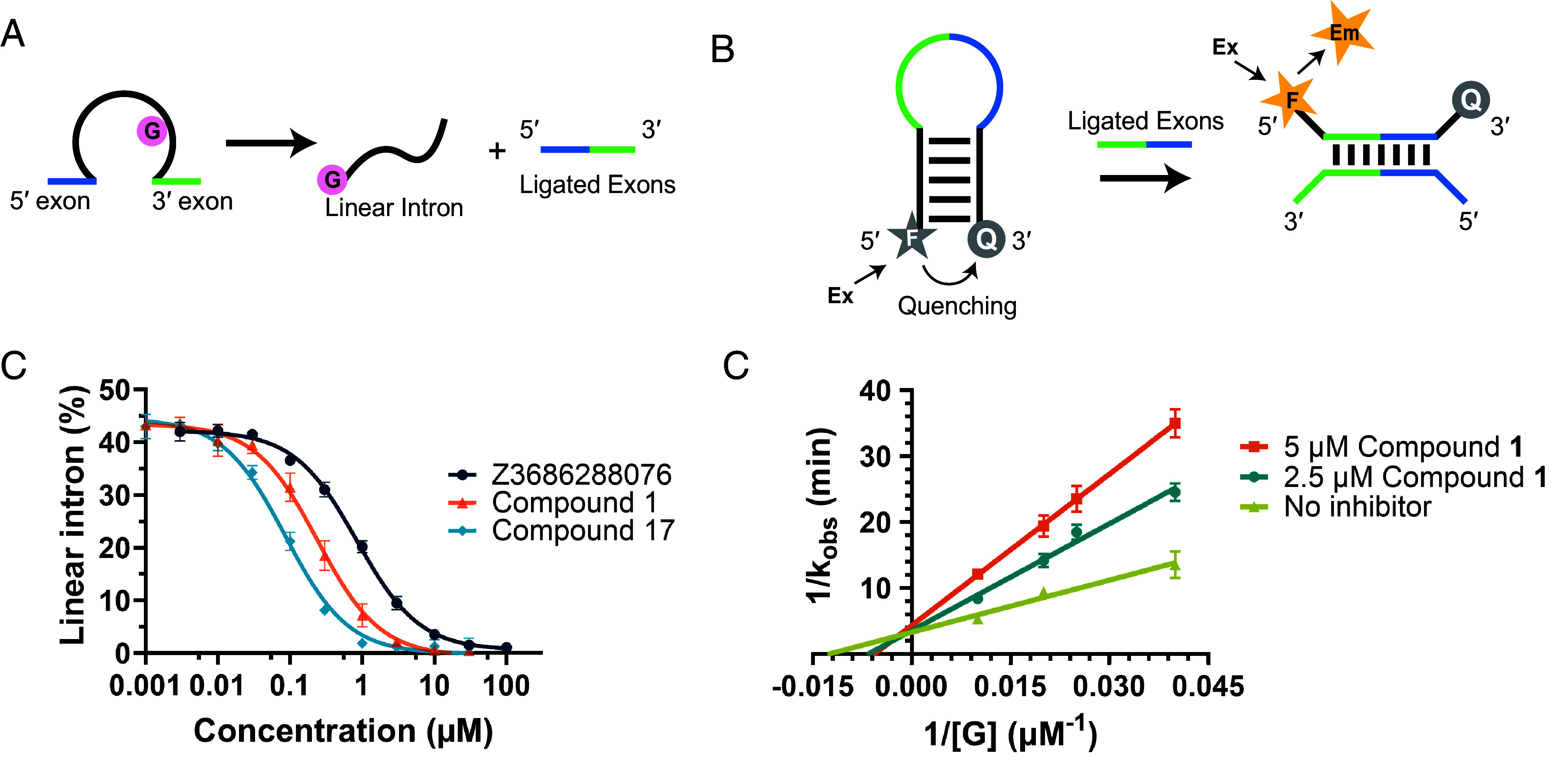
Identification and characterization of the group I intron inhibitor hit. (*A*) Cartoon of the group I intron splicing reaction. (*B*) Cartoon of the molecular beacon assay used for HTS (Ex - excitation, Em - emission, F - fluorophore, Q - quencher). (*C*) Dose-dependent splicing inhibition curves of selected compounds. A sensitive region of the splicing time course was used (~45% reaction completion at 10 min at 25 °C) in order to best compare the relative affinities of the compounds. Error bars represent SE of n = 2 technical replicates. (*D*) Lineweaver–Burk plot of compound **1** reveals the competitive mode of inhibition. Error bars represent SE of n = 2 technical replicates.

During previous studies, we identified and characterized a highly reactive self-splicing group IA1 intron that is found within the mitochondrial rRNA of *Candida albicans* (C.a.mtLSU) (31), representing a potential pan-fungal drug target due to its wide presence in diverse species of ascomycetes pathogenic fungi. To identify compounds that inhibit splicing of this intron (Fig. 1*A*), we implemented a high-throughput chemical screen that employs a molecular beacon, which specifically recognizes the ligated exon sequence generated by self-splicing of the *C. albicans* intron and thereby provides a sensitive fluorescent readout of splicing outcome (Fig. 1*B*). We employed this assay platform to screen for inhibitors within large compound libraries, obtaining the best results with the Enamine RNA-focused library (0.2% apparent hit rate) (*SI Appendix*, Fig. S1). A dose–response radioanalytic assay was then employed to filter out false positive hits due to fluorescence artifacts and to evaluate inhibition potency. Specificity of validated hits was tested by examining their effects on self-splicing of a structurally and mechanistically distinct group II intron system (32).

**Fig. 2.**
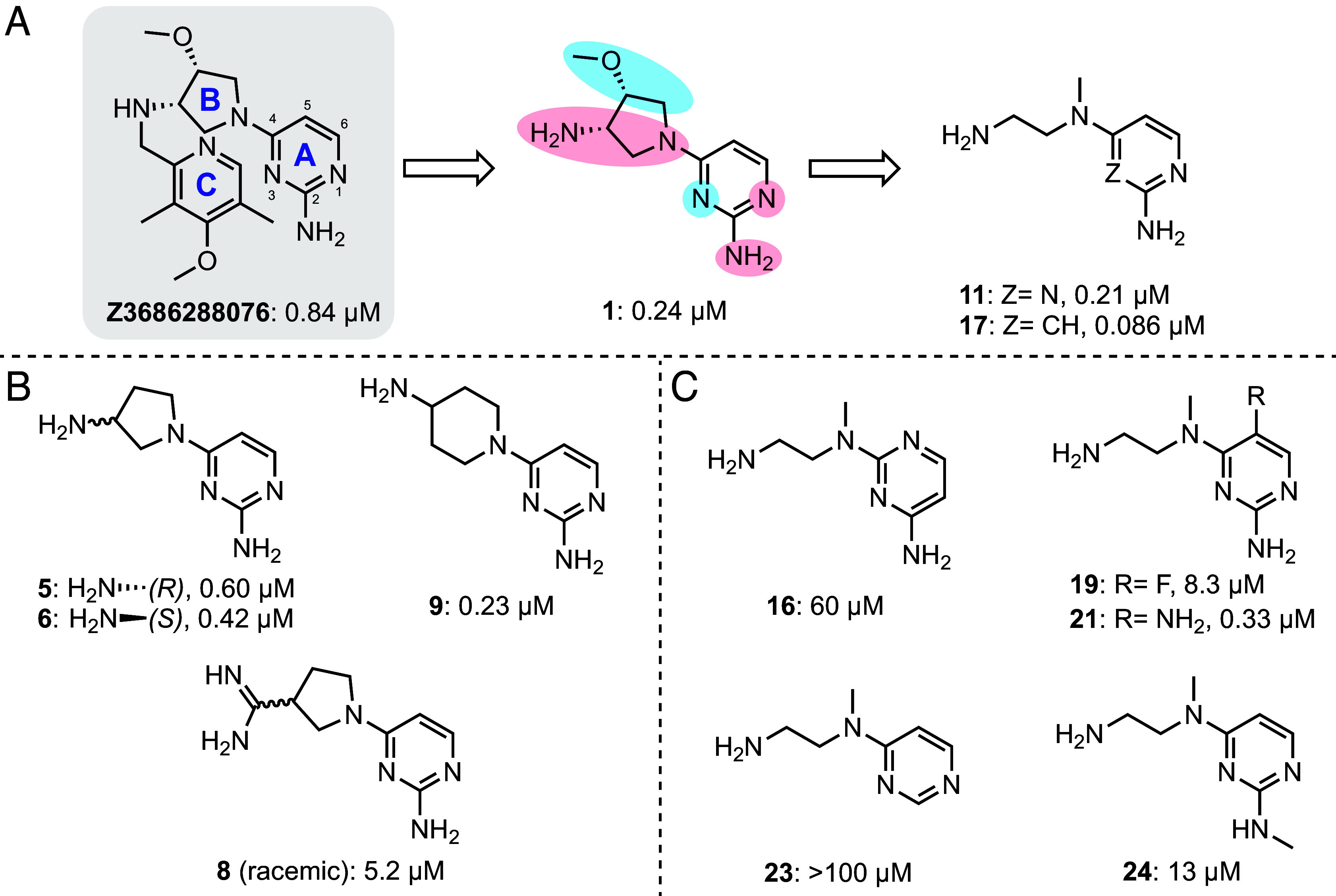
Summary of the SAR analysis. IC50 values for the inhibitor compounds are shown beneath each structure. (*A*) Optimization route with key compounds shown. Three ring structures in the initial hit (Z3686288076) are denoted as A, B, and C respectively. Initial hit expansion yielded compound **1** with ring C omitted. Red shading indicates portions of the molecule that are essential for activity, while blue indicates those not required. Further optimization led to compounds **11** and **17**. (*B*) Representative ring B modifications. (*C*) Representative ring A modifications.

A particular screen hit (Enamine catalog Z3686288076) was identified as a modestly potent and selective inhibitor of intron self-splicing (IC_50_ of 0.84 µM) (Fig. 1*C*). Given its modular scaffold architecture and synthetic tractability, Z3686288076 was selected for systematic evaluation. Preliminary structure–activity relationship (SAR) studies established that the pyrimidine conjugated with pyrrolidine, compound **1**, contained the pharmacophore for inhibition (Fig. 2*A* and *SI Appendix*, Table S2). To elucidate the mechanism of action, intron splicing rate was monitored as a function of compound **1** and guanosine cofactor, which is the nucleophile required for activity of all group I introns. Lineweaver–Burk plots of the resulting data demonstrate that compound **1** is a competitive inhibitor, with K_i_ of 0.67 μM (Fig. 1*D*), suggesting that the inhibitor binds within the conserved guanosine binding site.

### Potent Splicing Inhibitor Yielded from Hit Expansion.

To dissect the molecular determinants contributing to the potency of compound **1**, we carried out a detailed SAR study on this scaffold (Fig. 2, *SI Appendix*, Table S2), using a radioanalytical splicing assay as previously described (33). We first set out to determine whether the stereochemistry and cyclic nature of ring B were important for aligning atoms that contribute to intron recognition. To that end, we synthesized all four stereopairs of the amino and methoxy groups on ring B, finding that both *cis* isomers maintained inhibitory activity (compounds **1**–**4**, *SI Appendix*, Table S2). We then tested whether the oxygen substituent on ring B and the absolute stereochemistry of the primary amine contribute to function and found these changes have minimal impact on inhibition activity (compounds **5** and **6**, Fig. 2*B*, and *SI Appendix*, Table S2). By examining compounds containing alternative ring B structures (compounds **9** and **10**, Fig. 2*B*, and *SI Appendix*, Table S2), it became clear that the ring itself does not contribute to efficacy. However, the critical importance of the primary amine was evident when it was replaced with other functionalities, such as hydroxyl or amidine or secondary amine groups (compounds **7, 8,** and **14**, Fig. 2*B* and *SI Appendix*, Table S2).

Given these observations, we introduced a simple ethylene linker connecting the terminal and bridging nitrogen atoms to replace the original ring B design, resulting in compound **11**, which was chosen for subsequent structural studies of this hit series due to its high potency (*vida infra*). The importance of the tertiary amine and its relative positioning were probed via synthesis of modified analogs (compounds **12** and **13**). Similarly, to understand the role of substituents on ring A, we replaced heteroatoms at positions 1 and 3 with carbon, establishing the importance of N1 (compounds **16** and **17**, respectively, Fig. 2*C* and *SI Appendix*, Table S2). We note that the electronic properties of N1 are influenced by other substituents on ring A. For example, incorporation of a heterocyclic nitrogen or electron-withdrawing groups at the 5-position diminishes potency (compounds **18** and **19**, Fig. 2*C* and *SI Appendix*, Table S2), while addition of electron-donating groups at the same position maintains potency (compounds **20** and **21**, Fig. 2*C* and *SI Appendix*, Table S2). Moreover, the 6-position is intolerant to additional substituents (compound **22**). The importance of the amine substituent at the 2-position was further established by its deletion, or methylation (compounds **23** and **24**, Fig. 2*C* and *SI Appendix*, Table S2). Taken together, the substituent scan reveals a small, potent inhibitor scaffold and suggests functional groups that are likely to contribute to key interactions with the intron active site.

### CryoEM Elucidation of the Intron Bound to the Guanosine Cofactor.

**Fig. 3.**
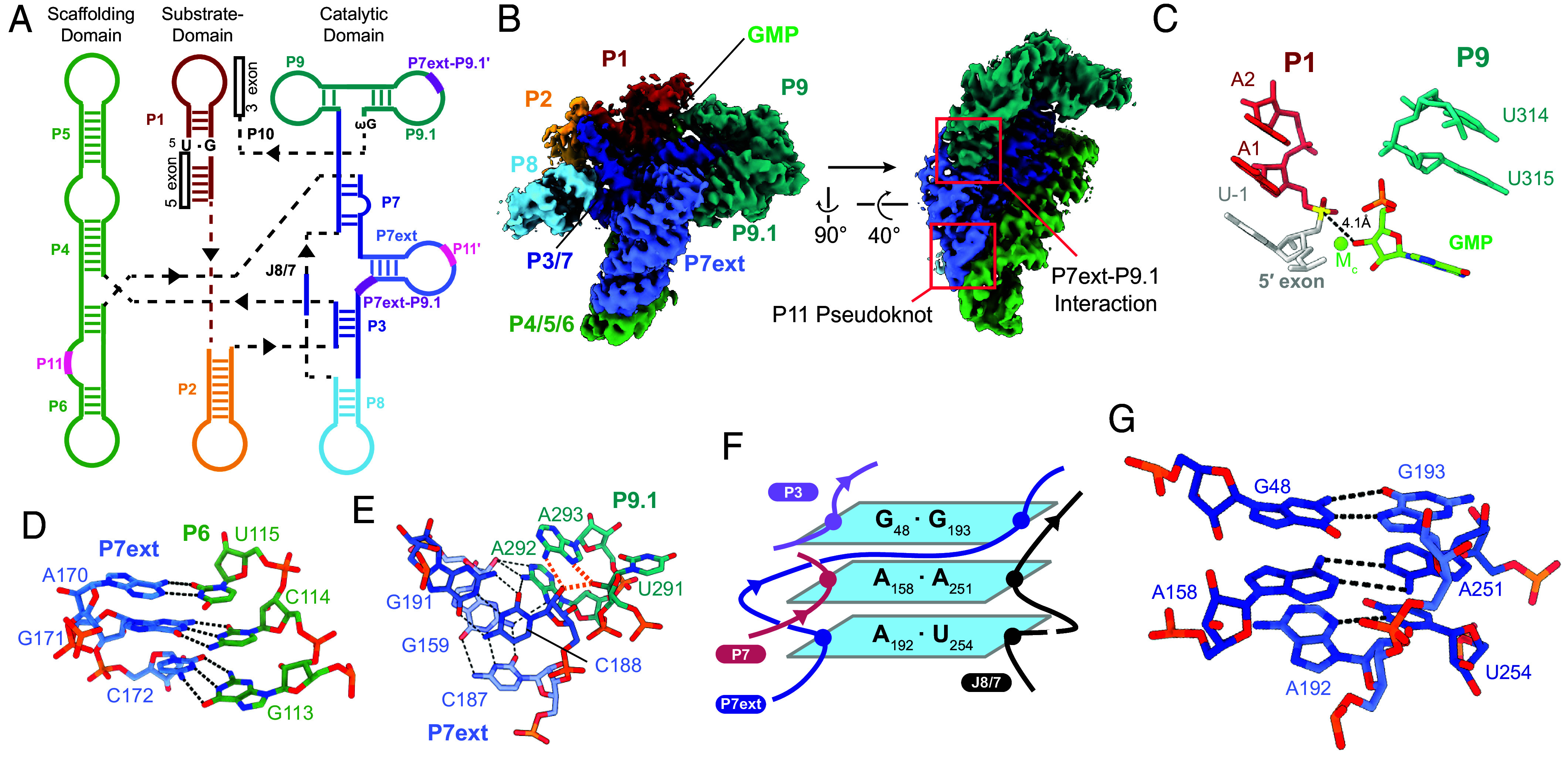
CryoEM structure of the *C. albicans* group I intron bound to GMP. (*A*) Secondary structure cartoon of the group I intron. In addition to the main helices (indicated as P1-P10), pseudoknot helix P11 (indicated with thick pink bars as P11 and P11′) and tertiary interactions P7ext-P9.1 (indicated with thick purple bars in the same manner) are illustrated. (*B*) CryoEM reconstruction of the group I intron with GMP bound. (*C*) Atomic model of the 5′ splice site prior to the first step of splicing. The GMP ligand is adjacent to the scissile phosphate (yellow). Dashed lines indicate the distance measurement between the attacking nucleophile and the scissile phosphate (4.1 Å). (*D*) Details of the P11 pseudoknot interaction and the specific base pairing between the P7ext and P6 helices. Dashed lines indicate base-pairing interactions. (*E*) Nucleotide contacts that form the P7ext-P9.1 tertiary interaction. Dashed lines indicate base-pairing interactions. Orange dashes highlight hydrogen bonding interactions involving A293. (*F* and *G*). Cartoon schematic and atomic model of the long-range base-pairing stacking sandwich between P3, P7, P7ext, and J8/7. Dashed lines indicate base-pairing interactions.

Before embarking on structural studies of the hit series, it was important to first establish the molecular interaction network between the C.a.mtLSU intron and guanosine itself using cryoEM (Fig. 3*A* and *SI Appendix*, Fig. S2). Previous genetic and structural studies have examined interactions between guanosine and group I introns (30, 34), but there have been no investigations of the unique group IA1 class. Here, we used calcium as the metal ion (32, 35) to trap the unspliced precursor RNA (the intron flanked by intact exons) bound to guanosine monophosphate (GMP). After freezing and imaging this complex, we observed an abundance of well-distributed particles, which were amenable to structure determination (*Materials and Methods*, *SI Appendix*, Fig. S2). From this dataset, we obtained a 3.1Å reconstruction (Fig. 3*B*) and built the atomic model of the intron RNA containing the bound guanosine ligand. In the structural model, the 3′-OH on bound guanosine, which serves as the reaction nucleophile, is close to the scissile phosphate with the corresponding catalytic metal M_C_ in place (Fig. 3*C*) (36, 37), indicating that the visualized structure is near the precatalytic state prior to the first step of splicing.

The cryoEM reconstruction revealed a canonical 3D arrangement for group I intron structures (38–40) with three main coaxially stacked RNA helices that encompass the intron core (Fig. 3*A*). However, in this case, we also visualized two distinctive structural modules (P7ext and P9.1) that brace the intron active site, which provide extensive mechanical support to the intron core and potentially explain the unusually robust biochemical activity of the C.a.mtLSU intron (31). One of these interactions is the P11 pseudoknot (31, 41), which anchors the P7ext helix against the extended P6 stem, effectively stapling the structural and catalytic domains of the intron together (Fig. 3*D*). Supporting this pseudoknot is a tertiary interaction between P9.1 and P7ext mediated by A-minor motifs (Fig. 3*E*) in which a network of 2′-OHs form long-range interactions with the nucleobase of A293. Finally, a unique stacking sandwich featuring noncanonical base pairs from four different RNA strands (P3, P7, P7ext, and J8/7) interdigitates the catalytic helices (Fig. 3 *F* and *G*). This unique structural module involving P7ext not only leads to a noncanonical P7 secondary structure for group I introns but also strengthens the association of multiple catalytic core elements by virtue of the stacking platform, highlighting the unique architectural role of P7ext as a hub for coordinated long-range interactions.

### CryoEM Elucidation of the Intron–Inhibitor Complex: Insights into Molecular Recognition.

**Fig. 4.**
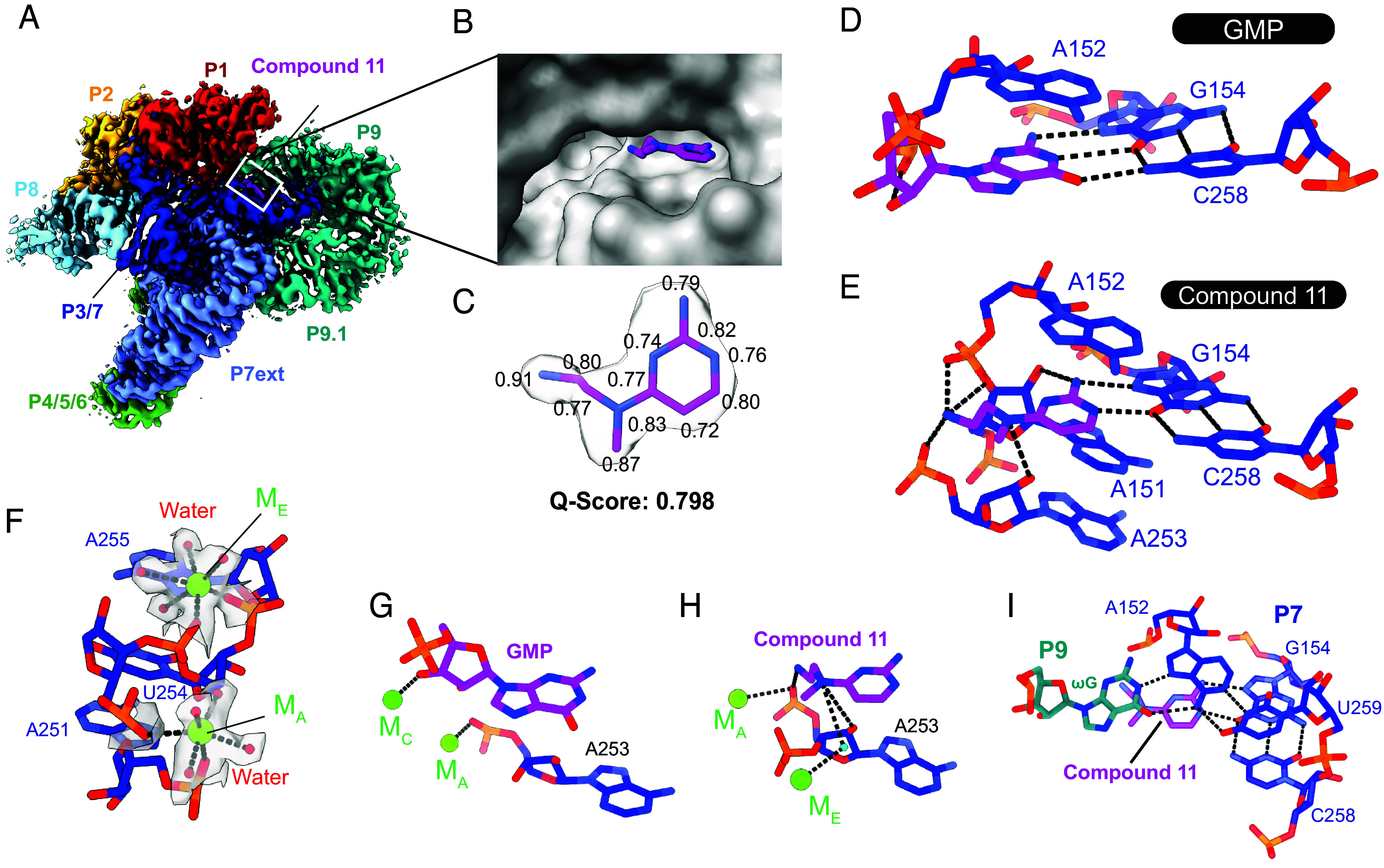
Structural basis of ligand recognition in the group I intron active site. (*A*) CryoEM density of the group I intron–compound **11** cocomplex. (*B*) Binding pocket within the active site occupied by the inhibitor. (*C*) Atomic model of compound **11** and the surrounding density. Q-scores of the individual atoms are indicated. The overall Q-score of the ligand is 0.798. (*D* and *E*) Details of molecular recognition of GMP and compound **11** within the group I intron active site. Hydrogen bonding interactions and metal coordination are shown as dashed lines. (*F*) Metal ion clusters within the group I intron core. Magnesium ions (green) and water molecules (red) are shown within their electron densities. Dashed lines represent metal coordination. (*G* and *H*) Metal ion coordination in the GMP- and compound **11**-bound structures. Dashed lines represent coordination with metal ions and hydrogen bonding (*I*) ωG-mediated base triple interaction with the P7 domain. Dashed lines represent hydrogen bonding interactions.

To gain molecular insights between the intron RNA and the hit series, we solved the structures of the intron in complex with compound **11** in the presence of either Mg^2+^ or Ca^2+^, achieving global resolutions of 2.4Å and 2.5Å respectively (Fig. 4*A* and *SI Appendix*, Figs. S3–S5). Comparison of the two reconstructions reveal minimal differences; hence we focused our analysis on the intron folded in Mg^2+^, which is of higher resolution (*SI Appendix*, Fig. S6).

We pinpointed a region of strong electron density (local resolution of 2.25Å) consistent with compound **11** in the pocket adjacent to intron P7 (Fig. 4 *B* and *C* and Movie S1). The quality of the density warranted confident placement of compound **11**. The Q-score (42) for the ligand was 0.79 (expected Q-score of 0.65 at 2.5Å), with the aminopyrimidine ring and nitrogen atoms in the ethylenediamine tail giving the strongest signal (Fig. 4*C* and *SI Appendix*, Fig. S7). No additional density indicative of nonspecific binding or alternative conformations was observed throughout the map, suggesting specificity of the compound to this highly structured RNA.

Comparing the binding of compound **11** (Fig. 4*D*) and GMP (Fig. 4*E*) unequivocally explains the structural basis for the competitive nature of compound **11** (*SI Appendix*, Fig. S8). The smaller 2-aminopyrimidine scaffold effectively mimics the guanine nucleobase, serving as a primary docking anchor by forming a similar base triple with the G154:C258 pair and stacking on A152. The interaction network is consistent with the SAR study, which showed that removal of the exocyclic amine or installation of an electron-withdrawing group that disrupts amine hydrogen-bonding potential, drastically reduces inhibition potency (compounds **23** and **19**, respectively). Second, the ethylenediamine tail of compound **11** functions as an additional tethering point, allowing it to outcompete guanosine for intron binding. The phosphates of A152 and A253 are intimately engaged with the terminal amino group (distance < 3Å) through both electrostatic and hydrogen bonding interactions, along with the bridging oxygen of A151 forming a hydrogen bond with the terminal amino group. These contacts are consistent with the SAR analysis showing that substitution of the terminal amine abolishes splicing inhibition activity (compound **7**). In contrast, guanosine has a weaker interaction network, engaging the phosphate of A152 through a single hydrogen bond with its 2′-OH. Finally, the bridging nitrogen atom of compound **11** engages the 2′-OH of A253, which is not involved in the case of guanosine. The extended interaction network we observed around compound **11** therefore makes it an effective competitor of GMP.

### Inhibitor-Induced Rearrangements within the Intron Active Site.

The high resolution of the inhibitor-bound RNA structure not only reveals an elaborate network of ligand recognition interactions, but it also enables us to visualize structural rearrangements that occur in response to the altered geometry and chemical composition of the bound ligand. Remarkably, we observed alterations in metal ion occupancy and coordination near the ligand-binding pocket (Fig. 4 *F*–*H*) (43). The catalytic metal M_C_, which is coordinated by the 2′- and 3′-OH of the guanosine ligand, is absent in the compound **11**-bound structure, likely due to the change in ligand geometry and electrostatic repulsion from the terminal amino group of the inhibitor. As a result, the second catalytic metal M_A_ moves slightly toward the position otherwise occupied by M_C_. In addition to the two catalytic metal ions, we could clearly see a structural metal M_E_ (44, 45) in the compound 11 structure located next to the phosphate of A253 in the compound **11**-bound structure, coordinating a water molecule. Intriguingly, the coordinated water participates in inhibitor recognition by forming a hydrogen bond with the bridging nitrogen atom of compound **11** (Fig. 4*H*). The functional consequence of this is supported by the fact that replacement of the bridging nitrogen with a hydrogen-bond-incompatible sulfur (compound **13**) is detrimental to inhibition activity. These observations comprise an unexpected strategy for RNA small-molecule recognition, whereby RNA-associated metal ions and their coordination sphere components exhibit plasticity, adapting in response to small-molecule ligands.

In a second example of ligand-induced plasticity, we observed a unique placement of the ultimate nucleotide of the intron, ωG (G316), within the inhibitor structure. The ωG nucleotide within a group I intron has never been visualized in positions other than the guanosine binding site (25, 30, 46, 47). As compound **11** is considerably smaller in size and presents less steric hindrance than guanosine, ωG enters a small cavity that is created by binding of the inhibitor, forming an unexpected base triple with the A152:U259 pair atop the ligand-mediated base triple (Fig. 4*I*). This altered arrangement within the guanosine binding pocket seals it shut, thereby limiting the dissociation of the bound inhibitor. Such entirely unexpected rearrangements underscore the striking complexity of small-molecule recognition by dynamic RNA tertiary structures.

### Dynamical Strategy for RNA–Ligand Recognition.

**Fig. 5.**
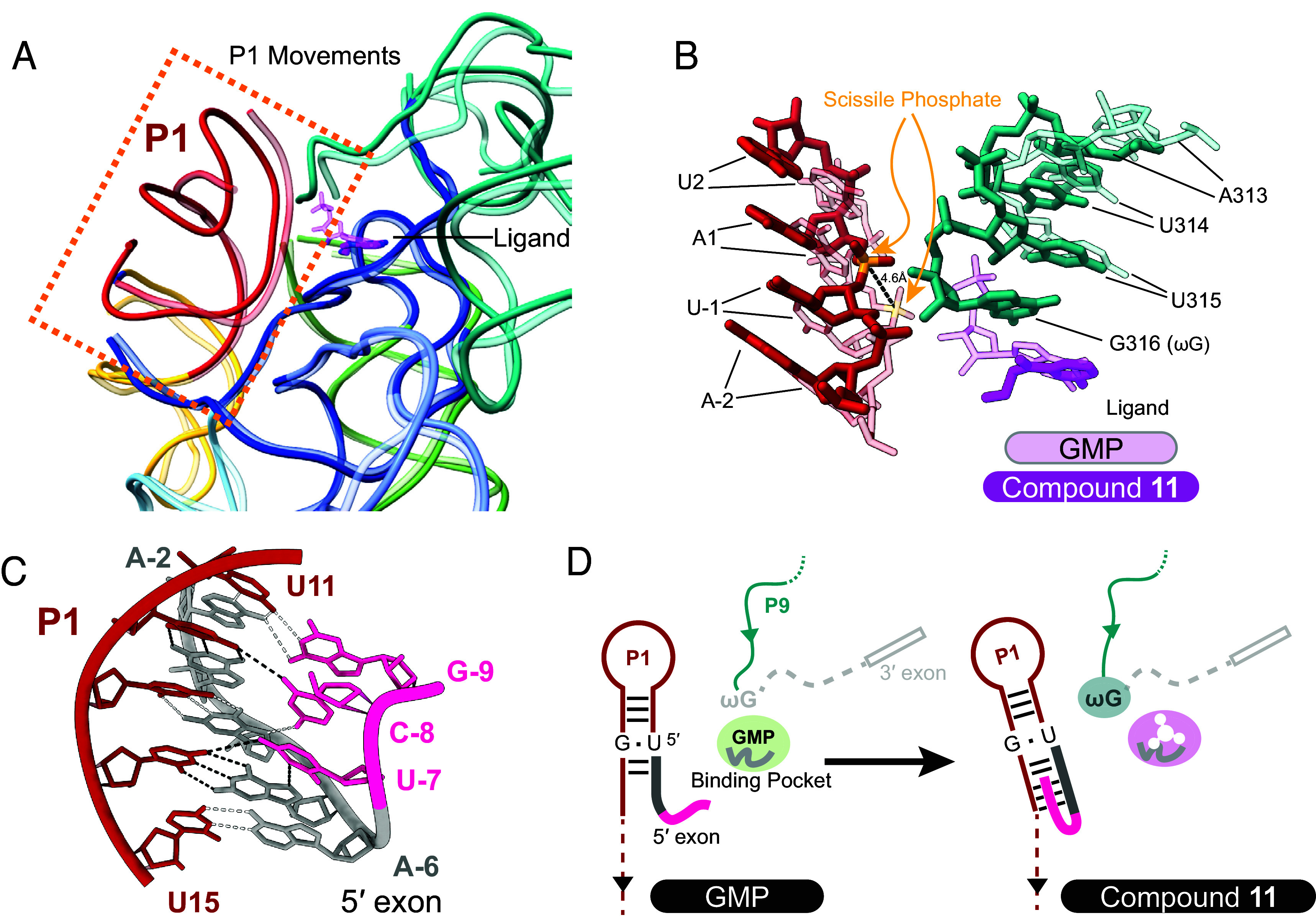
Dynamic RNA conformational changes upon compound 11 binding. (*A*) Comparison of the overall structure of the group I intron bound to GMP (colored in transparent) or compound **11** (colored in solid). The P1 helix moves outward when the inhibitor is bound. (*B*) Magnified overlay comparison of the 5′ splice site in the GMP and compound **11** atomic models. (*C*) Triple helix formed between the 5′ exon (shown in gray and pink) and the P1 helix. (*D*) Cartoon model of the dynamical consequences of compound **11** binding. Initially, GMP, the natural ligand of the intron binds. Once compound **11** is introduced it displaces GMP from the binding pocket, shifts the P1 stem away, ωG then occupies the newly freed space, and the triple helix between the P1 helix and the extended 5′ exon forms. The red color indicates the P1 helix. The 5′ exon is shown connected to the P1 helix as two segments in dark gray and pink as in **5C**. The teal color represents P9 of the intron which is linked to ωG and the 3′ exon, shown as a short rectangular box. The light gray shade indicates components of the intron not seen in the cryoEM reconstruction.

As RNA molecules often depend upon conformational plasticity for function (26, 48), we asked whether compound **11** binding induces conformational changes within the intron RNA beyond the immediate binding pocket. Through comparison of the two liganded structures of the intron RNA, we showed that the overall structural fold of the intron is preserved while the largest conformational change involves the P1 helix, which consists of the 5′-exon and the intronic internal guide sequence (IGS) (Fig. 5*A*). In the compound **11** complex, the ωG proximal to the ligand binding pocket appears to act as a gatekeeper, pushing on the upper stem of P1 and moving the scissile phosphate away from the intron catalytic core by 4.6 Å (Figs. 4*I* and 5*B*). Two hydrogen bonds originating from the 2′-OHs of A (-2) and G (-3) that contribute to normal P1 docking (49, 50) become disengaged as a result (*SI Appendix,* Fig. S9). By contrast, the basal stem of P1 is held in place by a network of interactions specific to the IA1 intron.

In addition to P1 displacement, we observed a special configuration of the P1 helix upon compound **11** binding. A sharp backbone kink develops between A (-6) and U (-7), allowing the extended 5′-exon to fold back and form the 5′-exon-IGS helix and giving rise to a short triple-helix within P1 (Fig. 5*C*), representing a unique strategy for the intron to recognize the extended 5′-exon beyond canonical base pairing. This particular arrangement appears to rigidify the conformation of the basal stem, neutralize the destabilizing effect of the disengaged upper stem, and prevent complete P1 disengagement as seen in previous structures of the *Tetrahymena* intron (51). Taken together, these observations reveal RNA conformational plasticity upon binding of a de novo ligand, revealing that recognition can be guided by a hybrid response that includes lock-and-key recognition, accompanied by induced fit strategies (Fig. 5*D*).

## Discussion

In this study, we demonstrate that chemical screening of a commercial RNA-focused library followed by SAR analysis and optimization of the resulting hit has led to the identification of a potent and selective group I intron splicing inhibitor. Through subsequent cryoEM studies, we were able to visualize high-resolution details of the molecular interaction network between the inhibitor and its group I intron RNA target (Movie S1). The resolution of 2.4Å enabled confident model building of the ligand and mapping of the molecular contacts, revealing unanticipated RNA dynamical strategies for ligand recognition. SAR derived from the medicinal chemistry campaign not only validated the cryoEM structural observations but also represents a fully structurally rationalized SAR that is unique in the RNA-targeting field.

From the medicinal chemistry perspective, the identification of our group I intron inhibitor hit series extends the guiding principles for designing RNA-targeting ligands generalized by efforts such as the R-BIND database (9–11). Indeed, the basic pharmacophore, consisting of the aminopyrimidine ring and ethylenediamine tail, features a high nitrogen content. From the SAR study and subsequent structural elucidation, nearly all nitrogen atoms in the ligand make key interactions to the molecular recognition network. By contrast, the methoxy group present in the initial hit is dispensable. The observations from our case study are therefore fully supportive of the nascent design principles that are emerging within the RNA-targeting chemical space (1, 5, 9, 10, 15).

The cryoEM structural analysis described in this study sheds light on ligand recognition mechanisms that are not otherwise obvious from the SAR observations. In addition to canonical hydrogen bonding and electrostatic interactions between compound **11** and the RNA molecule, the structural analysis shows that RNA-associated metal ions and their coordination sphere constituents, such as water molecules, play vital roles in adapting to ligand binding. RNA conformational dynamics add another layer of complexity to the ligand recognition strategy used by RNA molecules (48), which is reflected in this study as well. It is notable that the rigid macromolecular scaffold of the intron tertiary structure can still accommodate local rearrangements and flexibility near the ligand binding site, as exemplified by the unique ligand gating pose employed by the ωG, and P1 helix displacement, all of which contribute to ligand recognition and subsequent biochemical inhibition.

From a broader perspective, our findings represent important contributions to the databases being developed for virtual small-molecule screening and for RNA structure prediction. For example, the parallel structural and quantitative affinity metrics we provide on specific molecular interaction networks are particularly valuable, as they represent the type of foundational information that is needed for meaningful in silico screening against RNA targets. Likewise, successful in silico RNA structure prediction will become more accurate by incorporating the empirical data we provide in this study, including high-resolution spatial information on specific tertiary interaction networks, conformational sampling, and RNA structural responses to ligand binding. This is exemplified by the behavior of the P1 helix, which adopts alternate poses in response to different ligands (e.g., compound **11** and guanosine), underscoring the need to account for RNA conformational flexibility (48) during modeling and screening. In addition, we observe that divalent metal ion position and coordination environment exhibit plasticity that contribute to the specificity of ligand binding, suggesting that this type of effect should be considered for accurate docking prediction. Accounting for the full repertoire of RNA structural motifs, their dynamical motions, ligands, and cofactors will expand the database of RNA structural behavior beyond descriptors of discrete molecular interactions, ultimately increasing modeling accuracy and facilitating exploration of the vast chemical space to target the transcriptome.

In summary, our work represents a major milestone in the nascent RNA-targeting field. By combining innovative assays, a high-throughput splicing inhibitor screening platform, medicinal chemistry, and cryoEM visualization of liganded RNA structures, the work sets an important example in the field, which will inspire more efficient hit identification and structure-based drug design and contribute to the development of RNA-targeting small-molecule therapeutics.

## Materials and Methods

### In vitro Splicing Inhibition Assay.

Validated screen hits and their commercial analogs were purchased from Enamine. Optically pure compounds and additional synthetic analogs were synthesized and purified in-house at New England Discovery Partners (NEDP) (*SI Appendix*, *Biochemical Methods*). Powders were dissolved in DMSO to a final compound concentration of 10 mM for in vitro biochemical experiments.

In vitro splicing inhibition assays were performed by heat denaturing the self-splicing precursor RNA (10 nM final concentration) in a 50 mM K-HEPES pH 7.5 buffer at 95 °C for 1 min and cooling down at room temperature for 2 min. The intron RNA was refolded in 150 mM KCl and 3 mM MgCl_2_ for 5 min. 1 μL of compound stock solutions (20x concentration in DMSO) was added to the desired final concentration, leading to 5% (v/v) final DMSO% in the reaction mixture. Reaction was initiated by adding 10x guanosine stock solution to the final concentration of 20 μM and incubated at room temperature. Time point samples were taken and analyzed on 5% polyacrylamide denaturing gels as previously described (31).

Before undertaking IC50 analyses, we conducted splicing time courses and measured observed rate constants (*k*_obs_) for splicing inhibition at 10 µM compound. Time point samples of 0, 1.5, 3, 5, 7.5, 10, 20, 30, 45, and 60 min were taken, and data were fitted into the following single-exponential decay equation: Y = (Y_0_ - Y_Plateau_)*exp (-*k*_obs_*X) + Y_Plateau_, where Y_0_ = Initial precursor (%), Y = Precursor (%), and X = Time (min).

Compounds with *k*_obs_ values below 0.05 min^−1^ (55% of the DMSO control under the same conditions, *k*_obs_ = 0.09 ± 0.01 min^−1^) were subject to IC50 determination (except **3**, **4**, **7**, **13**, **15**, **16**, **18**, **22**, **23**, and **24)**, to obtain upper-bound IC50 values for compounds with low potency. A serial dilution of compound stocks was added to the folded intron precursor solution (2 nM final concentration) and incubated at 25 °C for 10 min. 20 µM guanosine was added to initiate the splicing reaction. All assays were performed in triplicate. The data were fitted into the 3-parameter logistic function: Min + (Max – Min)/(1 + ([I]/IC_50_), where [I] is the inhibitor concentration, Min is the fitted minimum response, Max is the fitted maximum response and IC_50_ is the half-maximal inhibitory concentration.

For the Lineweaver–Burk analysis, the inverse of observed rate constant (1/*k*_obs_) was plotted against the inverse of the substrate (guanosine) concentration (1/[G]) and fitted into a simple linear regression equation. The intersection of the double reciprocal plots of reactions performed at two compound **1** concentrations (2.5 and 5 µM), and of uninhibited reactions, were used to determine the mode of inhibition (52).

### Intron–Ligand Cocomplex Formation and Purification.

For structural studies, the self-splicing RNA construct of the *C.a.*mtLSU intron with A9U mutation (for stabilization of the P1 stem) and 40-nt each flanking exons was used (transcribed from BamHI-linearized plasmid pLTS228). The RNA was first heat denatured at 95 °C for 1 min and refolded in a buffer containing 50 mM K-HEPES pH 7.5 and 10 mM MgCl_2_ (for Mg^2+^ sample) or 10 mM CaCl_2_ (for Ca^2+^ sample) at 37 °C for 20 min. Refolded RNA was subsequently purified on an AKTA pure system with a Superdex 200 Increase 10/300 GL column (GE Healthcare) using the filtration buffer corresponding to the ionic condition used (50 mM K-HEPES pH 7.5 and 10 mM MgCl_2_; or 50 mM K-HEPES pH 7.5 and 10 mM CaCl_2_). The peak fraction was collected and the RNA concentration was measured on a NanoDrop spectrophotometer (Thermo). Purified intron RNA was then coincubated with aqueous stocks of either GMP or compound 11 to a final RNA concentration of 1 μM and a final ligand concentration of 5 mM.

### Grid Preparation and Data Collection.

For the intron–GMP complex (A9U RNA mutant), 3 μL of solution was applied to glow-discharged (30 s, 25 mA, 0.36 mBar) 200-mesh R2/2 Quantifoil Cu grids with 2 nm carbon coating. The grids were blotted for 2 s in 100% humidity at 4 °C with a blotting force offset of −4 and rapidly frozen in liquid ethane using a Vitrobot (Thermo Fisher). Grids were screened with a Glacios microscope operated at 200 keV and grids with good particle density and ice thickness were selected for data acquisition.

For the intron–compound **11** complex, 4 μL of the resulting solution was applied to glow-discharged (30 s, 25 mA, 0.36 mBar) 300-mesh R2/1 Quantifoil Cu grids with 2 nm carbon coating (EMS). The grids were blotted for 3 s in 100% humidity at 4 °C with a blotting force offset of −4 and rapidly frozen in liquid ethane using a Vitrobot (ThermoFisher). Grids were screened with a Glacios microscope (ThermoFisher) operated at 200 keV. Grids with good particle density and ice thickness were selected for data acquisition.

For cryoEM data collection, for the GMP complex, frozen grids were loaded into a Titan Krios microscope (FEI) operated at 300Â kV, equipped with a post-GIF K3 summit direct detection camera. A defocus range of −1 µm and −2.5 µm were used. Each micrograph was collected at 40 frames per second, 1.4115 e^−^/A^2^ dose per frame, a total exposure time of 1.075 s (43 frames), resulting in a total dose of 59.3 e^−^/A^2^. The nominal microscope magnification was 81,000x, corresponding to a physical pixel size of 1.068Â Å. 5,385 micrographs were collected using the SerialEM software, respectively.

For the compound **11** cocomplex, grids were loaded into a Titan Krios microscope (FEI) operated at 300 keV, equipped with a Falcon 4i direct detection camera (ThermoFisher), and the data were obtained in the counting mode. SerialEM v4.1.0 was used for data collection. Two datasets of 16,185 micrographs (Ca^2+^ sample) and 8,983 micrographs (Mg^2+^ sample) were collected. A nominal magnification of 165,000x and a defocus range of −0.5 μm to −2.0 μm was used, giving an effective pixel size of 0.743Å at the specimen level. Each micrograph was dose-fractionated to 40 frames with a total exposure time of 3.45 s, resulting in a total dose of 50 e^−^/A^2^.

### CryoEM Data Processing.

Recorded movie frames were processed using cryoSPARC v4.4.1 (53–55). Motion correction and CTF estimations were performed using default parameters in cryoSPARC. Exposures were curated and micrographs with obvious ice contamination, large motions, or broken regions were removed. For both the GMP and compound **11** intron complexes (Mg and Ca conditions), particles were initially picked using the automated blob picker and cleaned by iterative rounds of 2D classification. The filtered subset of particles was used for Topaz (56) training and picking, yielding 1,096,738 (GMP), 2,753,322 (Mg), and 2,314,797 (Ca) particles respectively. These particles were extracted with a box size of 256 × 256 pixels (GMP) and 320 × 320 pixels (compound **11;** Mg and Ca condition) respectively and binned twice for faster processing. An initial model was generated from a subset of 200 K, 500 K, and 250 K particles for the GMP, compound **11** – intron (Mg), and compound **11** – intron (Ca) structures respectively, which were then subjected to heterogeneous refinement, leaving subsets of 389,892, 612,762, and 316,599 particles. Next, particles were extracted to their full box sizes and multiple rounds of global and local CTF refinement, in addition to local refinement, were conducted to arrive at the final reconstructions. This yielded a map of 3.1Å for the GMP-intron cocomplex, 2.43 Å for the compound **11**-intron (Mg), and 2.59 Å for the compound 11-intron (Ca) as evaluated by the GSFSC with a cutoff of 0.143 (*SI Appendix*, Figs. S2, S3, and S5). All maps were aligned to the compound 11-intron (Mg) reconstruction. For visualization, final maps were autosharpened with Phenix 1.20.1 (57, 58) and displayed using PyMOL 2.5.4 and UCSF Chimera (59, 60).

### Model Building and Refinement.

To build the atomic structure of the *C.a.* mtLSU intron with GMP, the *Twort* virus group I intron (PDB: 1Y0Q) was superimposed into the postprocessed density map as the initial model. This was followed by manual adjustment and rebuilding with Coot 0.9.8.1 (61). Metal ions were manually placed using knowledge from previous crystal structures and according to the guidelines of metal ion assignments (62). This atomic model was subsequently used for docking into the compound **11** – intron cocomplex. The map density locally in the region of residues 309–311 is of lower resolution and local modeling may have some uncertainty (i.e., A261 may have an alternative conformation). The compound **11** chemical structure was generated from a SMILES file using eLBOW in Phenix. All maps and models were aligned and rescaled to the compound **11** – intron cocomplex (Mg). NAMDINATOR (63) (https://namdinator.au.dk/) was used for flexible fitting of the docked models to obtain better starting models for manual adjustment and rebuilding. Initial predicted secondary structure was refined based on the atomic model built into the reconstruction and the secondary structure diagram was manually drawn with Adobe Illustrator. The final models were improved by iterative rounds of real-space refinement against the sharpened cryoEM map in PHENIX (Phenix 1.20.1-4487) with subsequent rebuilding in COOT (57, 58, 61). Model building and validation statistics are listed in *SI Appendix*, Table S3.

## Supplementary Material

Appendix 01 (PDF)

Movie S1. **Group I Intron in complex with compound 11 (Supplemental to Figures 4B and 4C)**. This movie presents the cryo-EM structure of the group I intron bound to compound **11**, featuring both the density map and molecular model. The group I intron and compound **11** are color-coded as in Figure 4A for consistency. A 360° rotation provides an overview of the co-complex structure, followed by a focused view of the compound **11** binding pocket. In this region, strong electron density (magenta mesh) clearly defines the precise positioning of the compound **11** within the pocket. Structural visualization was performed using UCSF ChimeraX, as mentioned in the Methods section.

## Data Availability

CryoEM maps generated in this study are deposited in the Electron Microscopy Data Bank with codes EMD-48538 (GMP) (64), EMD-48539 (compound 11, magnesium) (65), and EMD-48540 (compound 11, calcium) (66). Structural models are available in the Protein Data Bank with PDB accession codes 9MQS (GMP) (67), 9MQT (compound 11, magnesium) (68), and 9MQU (compound 11, calcium) (69). All other data are included in the manuscript and/or supporting information.
