# Supplementary material for: Molecular insights into de novo small-molecule recognition by an intron RNA structure
Source: Proc Natl Acad Sci U S A. 2025 May 8;122(19):e2502425122. doi: 10.1073/pnas.2502425122 (PMC12088405; doi:10.1073/pnas.2502425122)
Supplement: Supplementary file 1 — Appendix 01 (PDF) [file pnas.2502425122.sapp.pdf]

Supporting Information for

Molecular insights into *de novo* small molecule recognition by an intron RNA structure

Tianshuo Liu[1, *], Ling Xu[1, 2, *, †], Kevin Chung[3, *], Luke J. Sisto[1,5], Jimin Hwang[1], Chengxin Zhang[1], Michael C. Van Zandt[5], Anna Marie Pyle[1, 2, 4, †]

Author affiliations:

[1] Department of Molecular, Cellular and Developmental Biology, Yale University, New Haven, CT 06511 USA.

[2] Howard Hughes Medical Institute, Chevy Chase, MD 20815, USA.

[3] Department of Molecular Biophysics and Biochemistry, Yale University, New Haven, CT 06511, USA.

[4] Department of Chemistry, Yale University, New Haven, CT 06511, USA.

[5] New England Discovery Partners, Branford, CT 06405, USA

* These authors contributed equally.

† To whom correspondence may be addressed: anna.pyle@yale.edu and ling.xu@yale.edu.

This PDF file includes:

Supporting text for **Materials and Methods**:
1. Biochemical Methods
2. Chemical Synthesis and Characterization

Figures and Legends S1 to S9
Legend for Movie S1
Tables S1 to S3
SI References

Other supporting materials for this manuscript include the following:

Movies S1 Group I Intron in complex with compound **11**

Supporting Information Text

Materials and Methods

1. Biochemical Methods

1.1 RNA Transcription and Purification

In vitro transcription of intron precursor RNA (without radioactive labelling) was performed as previously described(1). 40 µg of BamHI-linearized pLTS204 plasmid (for high-throughput screening and biochemical experiments) or pLTS228 plasmid (for structural studies, which contains an A9U mutation within the intron sequence for P1 stabilization) was added to each 1 ml of transcription and the reaction was incubated at 37°C for 6 hours. The reaction mixture was treated with DNase I (Invitrogen), ethanol precipitated and further purified on a 5% urea denaturing polyacrylamide gel to separate the band corresponding to the intron precursor RNA. The RNA was resuspended in a storage buffer (6 mM Na-MES pH 6.0) to a final concentration of 100 µM and frozen at -80°C until use.32P-body-labelled precursor RNA transcripts were prepared using 5 µg of linearized pLTS204 plasmid as previously described(32). The purified RNA was resuspended in the storage buffer to a final concentration of 200 nM and stored frozen until use.

1.2 Molecular Beacon Assay Reagents Preparation

DNA oligonucleotide TMB18 (5′-Am-CCAGG <u>TAC CCT GTT ATC CCT AGC</u> CCTGG-BHQ2-3′, loop sequence is underlined) containing the 3′-terminal Black Hole Quencher 2 (BHQ2) and 5′-terminal aminomodifier C3 TFA (Am) (Glen Research) were synthesized, deprotected and purified as previously described(2). Purified MB18 was labelled at the 5′-end aminomodifier with AlexaFluor 555 NHS ester (Life Technologies). The resulting dual-labelled molecular beacon was further purified on a 18% polyacrylamide (29:1) gel, eluted and ethanol precipitated. The pellet was resuspended in the ME buffer (8 mM K-MOPS pH 6.5, 1 mM EDTA) and stored at -80°C until use.

Positive control RNA oligonucleotide (5′- UAC GCU AGG GAU AAC AGG GUA AUA-3′) was synthesized by Integrated DNA Technologies (IDT) with standard desalting purification protocol.

1.3 Fluorescent Molecular Beacon Assay

The self-splicing precursor RNA of the *C.a.*mtLSU intron (transcribed from plasmid pLTS228) or the RNA target oligo in RNA storage buffer (6 mM Na-MES pH 6.0) was heated to 90°C for 1 min and was allowed to cool down to room temperature for 2 min. Wells of black 384-well plates (Corning 3575) were filled with 18 µl of solution containing 75 nM precursor RNA (containing 5 nM of radiolabeled precursor RNA) or the target oligo, 50 mM K-HEPES pH 7.5, 150 mM KCl, 3 mM $MgCl_2$, and 5% DMSO. Reaction was performed under room temperature and was initiated by the addition of 2 µl 10x guanosine aqueous solution (to the final concentration of 20 µM). Reaction was quenched by the addition of 2 µl EDTA solution (to the final concentration of 5 mM) at different points (for time 0, EDTA was added before guanosine). Three technical replicates were prepared for each time point. Then 2 µl of 10x molecular beacon solution was added (to the final concentration of 75 nM). The plate was heated at 70°C for 5 min, incubated on ice for 30 min and then room temperature for another 1 h before fluorescence reading on a Synergy Neo2 plate reader (Biotek) using a custom 540/590 filter (excitation wavelength: 540 nm and emission wavelength: 590 nm). 2 µl was taken from each well and mixed with 2 µl of 2x formamide loading dye for parallel radioanalytic analysis. The timepoint samples were then loaded onto a 5% denaturing polyacrylamide gel to resolve individual bands. The gel was dried and exposed to a phosphor storage screen. The screen was scanned on a Typhoon RGB biomolecular imager (Cytiva) using the phosphor-imaging functionality. Fraction of the ligated exon was calculated by dividing the normalized ligated exon band intensity (according to the uridine content in a given species) by the total intensity of three exon-containing bands (precursor, intron-3′exon intermediate and ligated exon).

Fluorescence reads at different time points were plotted as a bar chart in Prism 10.0 (GraphPad). The positive control involved incubation with RNA oligo and molecular beacon, while the negative control contained molecular beacon only. For the kinetic analysis, the fluorescence read or fraction of the ligated exon was plotted against time and fitted into a single-exponential accumulation equation in Kaleidagraph 4.5.4 (Synergy Software) to get the observed rate constants from both molecular beacon and radioanalytic methods.

1.4 High-throughput Screening

60 nl of compound stock solutions (10 mM in DMSO) from ECHO-qualified 384-well COC source plates (Labcyte LP-0200) were dispensed to black 384-well assay plates (Corning 3575) using the ECHO 550 acoustic dispensing system (Labcyte) to the final compound concentration of 30 µM. Columns 1, 2; 23, 24 were left empty for negative and positive controls respectively. Reaction master mix was then dispensed to the plates using a MultiDrop Combi dispenser (ThermoFisher) equipped with a small tube metal tip dispensing cassette (ThermoFisher) to the final assay condition of 50 mM K-HEPES pH 7.5, 150 mM KCl, 3 mM $MgCl_2$ and 5% (v/v) DMSO. RNA stock solution was first heat denatured at 90°C for 5 min, then cooled down at room temperature for another 5 min. 200 units of RNaseOUT (Invitrogen) was added to the RNA solution before dispensing to the plates (the final RNA concentration is 75 nM. The plates were then incubated at room temperature for 10 min to allow RNA folding. The reaction was initiated by dispensing the guanosine stock solution to the final concentration of 20 µM and incubated at room temperature for 30 min. The reaction was then quenched by dispensing the quench and readout mix (final concentration of 5 mM EDTA and 75 nM molecular beacon). The plates were incubated at 70°C for 5 min, incubated on ice for 30 min and then at room temperature for 1hr before fluorescence reading on a Synergy Neo2 plate reader with plate stacker (Biotek) using a custom 540/590 filter.

1.5 High-throughput Screening Data Analysis and Hit Validation

Compound inhibition percentage values were determined relative to the untreated wells (DMSO negative control, 0% inhibition) and to the wells lacking the self-splicing construct RNA (beacon only positive control, 100% inhibition), and inhibition percentage was calculated using Microsoft Excel. Screening scatter plot was generated in Prism 10.0 (GraphPad). The Z′-factor was calculated with the following equation(3): $Z' = 1 - 3 \times (\sigma_p + \sigma_n)/|\mu_p - \mu_n|$, where μ_p and σ_p are mean value and standard deviation for the positive control (molecular beacon only control), and μ_n and σ_n are the mean value and standard deviation for the negative control (DMSO control). The average Z′-factor from the screen was 0.78. A primary hit is defined as three standard deviations above the negative control (25% inhibition for the full screening set).

To validate the screen hits, compound stock solutions were cherry-picked from the screening library were dispensed in a dose series to black 384-well assay plates (Corning 3575) using the Echo 550 acoustic dispensing system (Labcyte). 5 nM of radiolabeled *C.a.*mtLSU precursor RNA was added to each well containing a reaction master mix consisting of 50 mM K-HEPES pH 7.5, 150 mM KCl, 3 mM $MgCl_2$ and 5% (v/v) DMSO. Reaction was initiated by addition of guanosine stock solution to the final concentration of 20 µM and the plates were incubated at room temperature for 20 min. 2 µl was then taken from each well and mixed with 2 µl of 2x formamide loading dye. The samples were then loaded onto a 5% denaturing polyacrylamide gel to resolve individual bands and analyzed as aforementioned. Compounds giving a dose-dependent inhibition of precursor conversion were defined as validated hits.

1.6 Competitive inhibition Analysis

For the Lineweaver-Burk analysis, the inverse of observed rate constant ($1/k_{obs}$) was plotted against the inverse of the substrate (guanosine) concentration (1/[G]) and fitted into a simple linear regression equation. The intersection of the double reciprocal plots of reactions performed at two compound **1** concentrations (2.5 and 5 µM), and of uninhibited reactions, were used to determine the mode of inhibition(1).

2. Chemical Synthesis and Characterization.

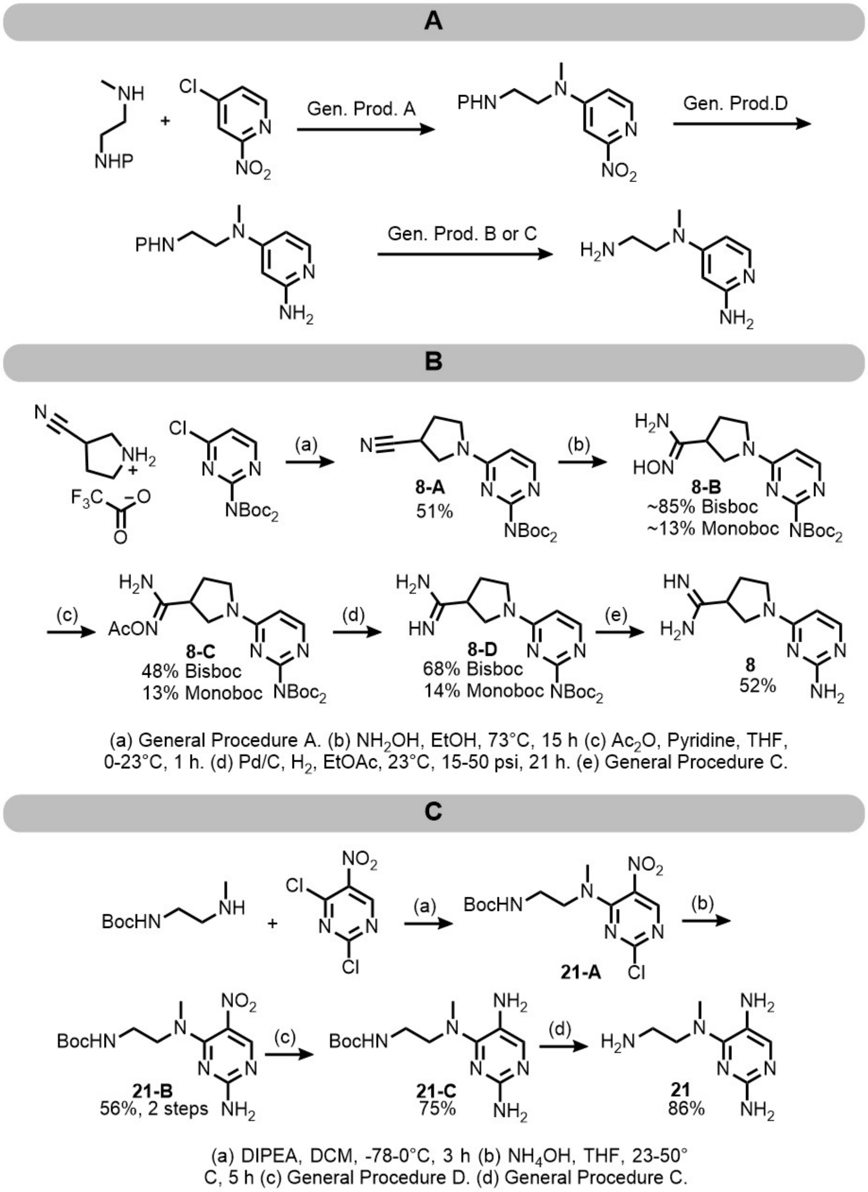

Scheme 1S: (A) General Scheme of Compound Synthesis. (B) Synthesis of 8. (C) Synthesis of 21.

All reactions were performed under a nitrogen atmosphere except for hydrogenations. Anhydrous solvents and 4M HCl in Dioxane were purchased from Sigma-Aldritch® (St. Louis, MS) and Fisher Scientific (Waltham, MA)®. Building blocks were obtained from either Combi-Blocks® (San Deigo, CA) or Ambeed® (Arlington Hts, IL). Palladium catalysts were purchased from TCI® USA. Additional reagents were sourced from TCI ® (Philadelphia, PA) and Oakwood Chemical® (Estill, SC). NMR Solvents were obtained from Cambridge Isotope Laboratories ® (Tewksbury, MA) and NMR spectra recorded on a Bruker® AdvanceCore 400 MHz spectrometer. HPLC analysis was performed on either an Agilent ® 1100 or 1260 system.

Abbreviations Used

ACN, acetonitrile

BOC, *tert*-butoxycarbonyl

Cbz, benzyloxycarbonyl

$CHCl_3$, chloroform

DBU, 1,8-Diazabicyclo(5.4.0)undec-7-ene

DCM, dichloromethane

DIPEA, N, N-Diisopropylethylamine

equiv., equivalents

EtOH, ethanol

HPLC, High-Performance Liquid Chromatography

IPA, isopropanol

M, molar

MeOH, methanol

MPLC, medium pressure chromatography

MsCl, methanesulfonyl chloride

NMR, Nuclear Magnetics Resonance

Pd/C, Palladium on carbon, 10% by weight

SnAR, Nucleophilic Aromatic Substitution

TEA, triethylamine

TFA, trifluoroacetic acid

TFAA, trifluoroacetic acid anhydride

THF, tetrahydrofuran

TLC, thin layer chromatography

vol., volumes

General Procedures: Many of the compounds described are synthesized using several common conditions. Synthetic procedures for compounds will refer to the general procedures used in their synthesis using the capital letters assigned to the steps below.

General Procedure A: SnAR of Heterocycles and Amines

To a glass pressure vessel equipped with stir bar was added the chloroheterocycle (1.0 equiv.) and the amine or derived HCl salt (1.0 equiv.). The reactants were suspended in an equal volume of EtOH and DIPEA to afford a 0.7 M solution. The pressure vessel was capped and heated at 100 °C for 16 hours with stirring. The reaction was then cooled to room temperature. If product precipitated the solid was filtered, rinsed with 2 reaction volumes of EtOH, and dried *in vacuo* to afford sufficiently pure product (>95% HPLC). If the product remained in solution the reaction solvent was removed *in vacuo* and the residue dissolved in 10 reaction volumes of DCM. The organic layer was washed with saturated aqueous $NaHCO_3$ (3x 3 vol.), brine (1x 3 vol.), dried over anhydrous $MgSO_4$, filtered, and the solvent was removed *in vacuo*. The residue was purified by MPLC using silica gel and a MeOH/DCM gradient.

HPLC conditions:

Column: Phenomenex Synergi: 4µm Polar-RP 80 Å 250 x 4.6 mm

Mobile Phase: A – water with 0.1% TFA, B – ACN with 0.1% TFA

Flow rate: 1.0 mL/minute

Gradient: 5-95% B from 0-10 minutes, 95% B from 10-13 minutes

General Procedure B: Cbz deprotection

A Cbz protected amine (1.0 equiv.) was placed in a round bottom flask with a stir bar and suspended in EtOH to afford a 0.165 M solution. The flask was purged with vacuum and backed filled 3x with N_2(g) before the addition of Pd/C (0.1 equiv. 10% by weight) and $Pd(OH)_2$/C (0.05 equiv. 20% by weight). A T-valve adapter equipped with a H_2(g) balloon was fitted and the reaction was purged with vacuum and backed filled (3x) with H_2(g) before stirring at 40 °C for 2.5 hours. After that time the reaction was cooled to room temperature and purged with vacuum before being backed filled 3x with N_2(g). The reaction was filtered through a Celite ® pad, and the pad was rinsed with 3 reaction volumes of MeOH. The filtrate was concentrated *in vacuo*, dissolved in MeOH and treated with 4 M HCl in dioxanes (2.2 equiv.). The solvent was removed *in vacuo* to provide product as a hydrochloride salt.

General Procedure C: Boc deprotection

A Boc protected amine (1.0 equiv.) was placed in a round bottom flask with a stir bar and suspended in 4 M HCl in dioxanes to afford a 0.15 M solution. The mixture was heated to 40 °C for 2.5 hours before cooling to room temperature. The solid was filtered off and rinsed with one reaction volume of THF to furnish product as a hydrochloride salt.

General Procedure D: Nitro deprotection

A nitroheterocycle (1.0 equiv.) was added to a degassed solution of MeOH and Pd/C (0.16 equiv. 10% by weight) to afford a 0.078 M solution. A T-valve adapter equipped with a H_2(g) balloon was fitted and the reaction was purged with vacuum and backed filled 3x with H_2(g) before stirring at 23 °C for 3.5 hours. After that time the reaction was purged with vacuum before being backed filled 3x with N_2(g). The reaction was filtered through a Celite ® pad, and the pad was rinsed with 3 reaction volumes of MeOH. The filtrate was concentrated *in vacuo* to provide product.

Synthesis of Intron inhibitors Using General Procedures

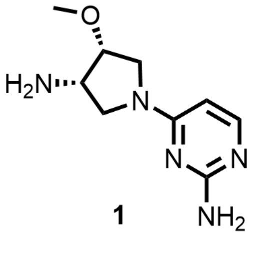

1

4-((3S,4R)-3-amino-4-methoxypyrrolidin-1-yl)pyrimidin-2-amine dihydrochloride (1 2HCl): This compound was synthesized using general procedures **A** and **B**, with yields of 76 and 85% respectively.

^{1}H NMR (400 MHz, Deuterium Oxide) δ 7.65 (d, J = 7.5 Hz, 1H), 6.20 (d, J = 7.5 Hz, 0.55 H), 6.17 (d, J = 7.5 Hz, 0.45H), 4.35 – 4.21 (m, 1H), 4.18 – 3.99 (m, 2H), 3.87 – 3.58 (m, 3H), 3.43 (s, 1.65H), 3.42 (s, 1.35H).

^{13}C NMR (101 MHz, Deuterium Oxide) 161.11, 161.10, 154.48, 154.46, 141.39, 141.37, 96.60, 96.50, 77.26, 76.52, 57.39, 57.22, 50.58, 50.02, 49.58, 49.44, 47.45, 47.38. All peaks visibly doubled (*vide infra*).

LCMS-ESI m/z calculated for $C_9H_{15}N_5OH^+$ [M+H$^+$] 210.13, found 210.2

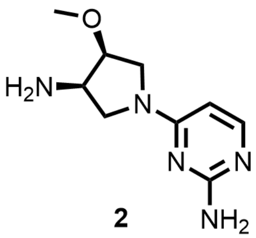

2

4-((3R,4S)-3-amino-4-methoxypyrrolidin-1-yl)pyrimidin-2-amine dihydrochloride (2 2HCl): This compound was synthesized using general procedures **A** and **B**, with yields of 86 and 69% respectively.

^{1}H NMR (400 MHz, Deuterium Oxide) δ 7.65 (d, J = 7.5 Hz, 1H), 6.20 (d, J = 7.5 Hz, 0.55 H), 6.17 (d, J = 7.5 Hz, 0.45H), 4.35 – 4.21 (m, 1H), 4.18 – 3.99 (m, 2H), 3.87 – 3.58 (m, 3H), 3.43 (s, 1.65H), 3.42 (s, 1.35H).

^{13}C NMR (101 MHz, Deuterium Oxide) 161.11, 161.10, 154.48, 154.46, 141.39, 141.37, 96.60, 96.50, 77.26, 76.52, 57.39, 57.22, 50.58, 50.02, 49.58, 49.44, 47.45, 47.38. All peaks visibly doubled (*vide infra*).

LCMS-ESI m/z calculated for $C_9H_{15}N_5OH^+$ [M+H$^+$] 210.13, found 210.3.

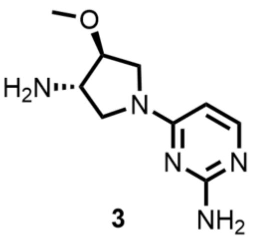

3

4-((3S,4S)-3-amino-4-methoxypyrrolidin-1-yl)pyrimidin-2-amine dihydrochloride (3 2HCl): This compound was synthesized using general procedures **A** and **B**, with yields of 67 and 94% respectively.

^{1}H NMR (400 MHz, Deuterium Oxide) δ 7.66 (d, J = 7.4 Hz, 1H), 6.20 (d, J = 7.4 Hz, 0.5H), 6.20 (d, J = 7.5 Hz, 0.5H), 4.35 – 4.19 (m, 1H), 4.15 – 3.93 (m, 3H), 3.90 – 3.60 (m, 2H), 3.43 (s, 1.5H), 3.43 (s, 1.5H).

^{13}C NMR (101 MHz, Deuterium Oxide) δ 161.18, 161.15, 154.50, 154.47, 141.56, 96.71, 96.60, 80.49, 79.76, 57.44, 57.33, 52.81, 52.17, 50.17, 50.08, 48.38, 48.16. All peaks visibly doubled except for 141.56 (*vide infra*).

LCMS-ESI m/z calculated for $C_9H_{15}N_5OH^+$ [M+H$^+$] 210.13, found 210.1.

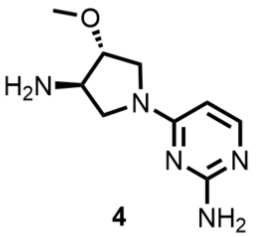

4

4-((3R,4R)-3-amino-4-methoxypyrrolidin-1-yl)pyrimidin-2-amine dihydrochloride (4 2HCl): This compound was synthesized using general procedures **A** and **B**, with yields of 73 and 53% respectively.

^{1}H NMR (400 MHz, Deuterium Oxide) δ 7.66 (d, J = 7.4 Hz, 1H), 6.20 (d, J = 7.4 Hz, 0.5H), 6.20 (d, J = 7.5 Hz, 0.5H), 4.35 – 4.19 (m, 1H), 4.15 – 3.93 (m, 3H), 3.90 – 3.60 (m, 2H), 3.43 (s, 1.5H), 3.43 (s, 1.5H).

^{13}C NMR (101 MHz, Deuterium Oxide) δ 161.18, 161.15, 154.50, 154.47, 141.56, 96.71, 96.60, 80.49, 79.76, 57.44, 57.33, 52.81, 52.17, 50.17, 50.08, 48.38, 48.16. All peaks visibly doubled except for 141.56 (*vide infra*).

LCMS-ESI m/z calculated for $C_9H_{15}N_5OH^+$ [M+H$^+$] 210.13, found 210.1.

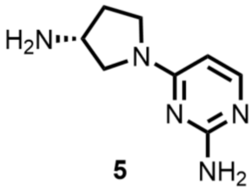

5

(R)-4-(3-aminopyrrolidin-1-yl)pyrimidin-2-amine dihydrochloride (5 2HCl): This compound was synthesized using general procedures **A** and **C**, with yields of 89 and 69% respectively.

^{1}H NMR (400 MHz, Deuterium Oxide) δ 7.72 (d, J = 7.5 Hz, 1H), 6.29 (d, J = 7.5 Hz, 0.55H), 6.25 (d, J = 7.5 Hz, 0.45H), 4.32 – 4.09 (m, 1H), 4.08 – 3.95 (m, 1H), 3.94 – 3.71 (m, 3H), 2.68 – 2.44 (m, 1H), 2.40 – 2.17 (m, 1H).

^{13}C NMR (101 MHz, D2O) δ 160.79, 160.65, 154.49, 154.43, 141.16, 96.83, 96.79, 50.30, 50.16, 50.01, 49.35, 44.98, 44.93, 28.76, 28.12. All peaks visibly doubled except for 141.16 (*vide infra*).

LCMS-ESI m/z calculated for $C_8H_{13}N_5H^+$ [M+H$^+$] 180.12, found 180.1.

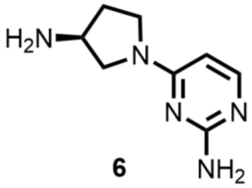

6

(S)-4-(3-aminopyrrolidin-1-yl)pyrimidin-2-amine dihydrochloride (6 2HCl): This compound was synthesized using general procedures **A** and **C**, with yields of 74 and 92% respectively.

^{1}H NMR (400 MHz, Deuterium Oxide) δ 7.72 (d, J = 7.5 Hz, 1H), 6.29 (d, J = 7.5 Hz, 0.55H), 6.25 (d, J = 7.5 Hz, 0.45H), 4.32 – 4.09 (m, 1H), 4.08 – 3.95 (m, 1H), 3.94 – 3.71 (m, 3H), 2.68 – 2.44 (m, 1H), 2.40 – 2.17 (m, 1H).

^{13}C NMR (101 MHz, D2O) δ 160.79, 160.65, 154.49, 154.43, 141.16, 96.83, 96.79, 50.30, 50.16, 50.01, 49.35, 44.98, 44.93, 28.76, 28.12. All peaks visibly doubled except for 141.16 (*vide infra*).

LCMS-ESI m/z calculated for $C_8H_{13}N_5$ H^+ [M+H$^+$] 180.12, found 180.1.

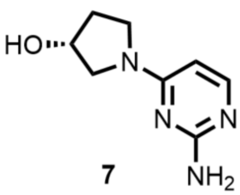

7

(**R)-1-(2-aminopyrimidin-4-yl)pyrrolidin-3-ol hydrochloride (7 HCl):** This compound was synthesized using general procedure **A** in 77% yield. The HCl salt was formed afterwards in quantitative yield by treating a 0.5 M methanolic solution of freebase with HCl (1.1 equiv., 4 M in Dioxanes).

^{1}H NMR (400 MHz, Deuterium Oxide) δ 7.65 (dd, J = 7.5, 4.4 Hz, 1H), 6.24 (dd, J = 19.6, 7.5 Hz, 1H), 4.71 – 4.58 (m, 1H), 4.09 – 3.48 (m, 4H), 2.44 – 2.01 (m, 2H).

^{13}C NMR (101 MHz, D2O) δ 160.40, 160.29, 154.34, 154.31, 140.46, 140.43, 96.97, 96.93, 70.01, 69.36, 55.04, 54.76, 45.37, 45.25, 32.57, 32.00. All peaks visibly doubled (*vide infra*).

LCMS-ESI m/z calculated for $C_8H_{12}N_4OH^+$ [M+H$^+$] 181.10, found 181.1.

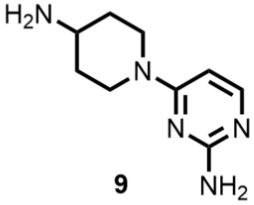

9

4-(4-aminopiperidin-1-yl)pyrimidin-2-amine dihydrochloride (9 2HCl): This compound was synthesized using general procedures **A** and **C**, with yields of 67 and 78% respectively.

^{1}H NMR (400 MHz, Deuterium Oxide) δ 7.68 (d, J = 7.7 Hz, 1H), 6.48 (d, J = 7.7 Hz, 1H), 5.01 (d, J = 12.6 Hz, 1H), 4.22 (d, J = 12.4 Hz, 1H), 3.70 – 3.53 (m, 1H), 3.45 – 3.22 (m, 1H), 3.18 – 2.92 (m, 1H), 2.41 – 2.11 (m, 2H), 1.85 – 1.57 (m, 2H). Piperidine is seemingly desymmetrized in ^{1}H and ^{13}C NMR spectra, freebasing eliminates this artifact and proves structure (*vide infra*).

^{13}C NMR (101 MHz, Deuterium Oxide) δ 161.47, 154.53, 141.58, 95.57, 47.84, 43.94, 41.87, 29.28, 28.99.

LCMS-ESI m/z calculated for $C_9H_{15}N_5H^+$ [M+H$^+$] 194.14, found 194.3.

4-(4-aminopiperidin-1-yl)pyrimidin-2-amine (9 Freebase): An analytical quantity **9** freebase (~10 mg) was obtained by dissolving **9** dihydrochloride (~15 mg) in 1 ml of methanol. Thoroughly rinsed Amberlyst ® A26 hydroxide resin was added until a pH of 10.5 was reached as indicated by Supelco ® pH strips. The reaction was filtered via syringe and the solvent removed *in vacuo* to furnish **9 Freebase**. The piperidine ring of the freebase was not desymmertrized in ^{1}H or ^{13}C NMR, indicating the desymmertrized dihydrochloride spectra were an artifact of salt formation. This artifact is responsible for the doubling ^{1}H and ^{13}C NMR peaks throughout the work. Cyclic structures and structures with linkers longer than ethylene demonstrate a greater level of splitting. Not all signals in a structure are split to the same, perceivable degree in ^{1}H and ^{13}C NMR spectra.

^{1}H NMR (400 MHz, Deuterium Oxide) δ 7.79 (d, J = 6.4 Hz, 1H), 6.22 (d, J = 6.5 Hz, 1H), 4.24 (d, J = 13.5 Hz, 2H), 3.08 – 2.88 (m, 3H), 1.99 – 1.77 (m, 2H), 1.40 – 1.15 (m, 2H).

^{13}C NMR (101 MHz, Deuterium Oxide) δ 162.55, 162.47, 155.77, 94.97, 47.68, 42.98, 33.42. Piperidine ring is not desymmetrized.

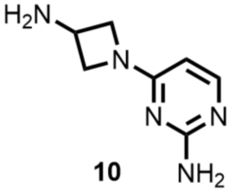

10

4-(3-aminoazetidin-1-yl)pyrimidin-2-amine (10 2HCl): This compound was synthesized using general procedures **A** and **C**, with yields of 75 and 84% respectively

^{1}H NMR (400 MHz, Deuterium Oxide) δ 7.69 (d, J = 7.3 Hz, 1H), 6.09 (d, J = 7.3 Hz, 1H), 4.74 – 4.60 (m, 2H), 4.48 – 4.29 (m, 3H).

^{13}C NMR (101 MHz, Deuterium Oxide) δ 161.96, 154.72, 141.32, 94.89, 54.39, 53.90, 40.66. Split Azetidine, ^{13}C NMR ~54.0 visibly doubled.

LCMS-ESI m/z calculated for $C_7H_{11}N_5H^+$ [M+H$^+$] 166.11, found 166.1.

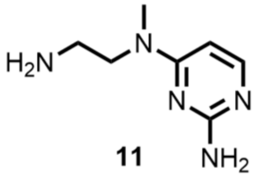

11

N4-(2-aminoethyl)-N4-methylpyrimidine-2,4-diamine dihydrochloride (11 2HCl): This compound was synthesized using general procedures **A** and **C**, with yields of 94 and 68% respectively.

^{1}H NMR (400 MHz, Deuterium Oxide) δ 7.73 (d, J = 7.6 Hz, 1H), 6.41 (d, J = 7.6 Hz, 1H), 4.05 (t, J = 5.8 Hz, 2H), 3.36 (t, J = 5.8 Hz, 2H), 3.23 (s, 3H).

^{13}C NMR (101 MHz, Deuterium Oxide) δ 163.79, 154.16, 141.70, 95.94, 46.92, 37.75, 36.39.

LCMS-ESI m/z calculated for $C_7H_{13}N_5H^+$ [M+H$^+$] 168.12, found 168.2.

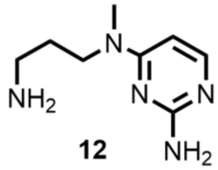

12

N4-(3-aminopropyl)-N4-methylpyrimidine-2,4-diamine dihydrochloride (12 2HCl): This compound was synthesized using general procedures **A** and **C**, with yields of 97 and 39% respectively.

^{1}H NMR (400 MHz, Deuterium Oxide) δ 7.73 – 7.54 (m, 1H), 6.45 – 6.26 (m, 1H), 3.90 – 3.58 (m, 2H), 3.26 – 3.12 (m, 3H), 3.11 – 2.93 (m, 2H), 2.17 – 1.97 (m, 2H).

^{13}C NMR (101 MHz, Deuterium Oxide) δ 162.71, 162.20, 154.32, 154.14, 141.08, 140.97, 95.86, 95.65, 47.75, 45.73, 36.82, 36.63, 35.98, 35.75, 24.79, 24.32. All peaks visibly doubled.

LCMS-ESI m/z calculated for $C_8H_{15}N_5H^+$ [M+H$^+$] 182.14, found 182.2.

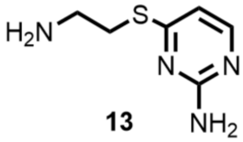

13

4-((2-aminoethyl)thio)pyrimidin-2-amine dihydrochloride (13 2HCl): This compound was synthesized by substituting the amine in general procedure **A** with the relevant thiol. The yields were 69 and 86% for general procedures **A** and **C** respectively.

^{1}H NMR (400 MHz, Deuterium Oxide) δ 7.89 (d, J = 6.9 Hz, 1H), 6.96 (d, J = 6.9 Hz, 1H), 3.58 (t, J = 6.3 Hz, 2H), 3.44 (t, J = 6.5 Hz, 2H).

^{13}C NMR (101 MHz, Deuterium Oxide) δ 179.89, 153.97, 141.61, 108.71, 38.61, 26.79.

LCMS-ESI m/z calculated for $C_6H_{10}N_4SH^+$ [M+H$^+$] 171.07, found 171.2.

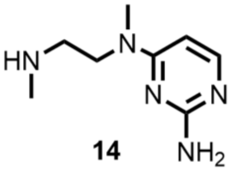

14

N4-methyl-N4-(2-(methylamino)ethyl)pyrimidine-2,4-diamine dihydrochloride (14 2HCl): This compound was synthesized using general procedures **A** and **C**, with yields of 91 and 79% respectively.

^{1}H NMR (400 MHz, Deuterium Oxide) δ 7.73 (d, J = 7.6 Hz, 1H), 6.40 (d, J = 7.6 Hz, 1H), 4.10 – 4.04 (m, 2H), 3.40 (t, J = 5.6 Hz, 2H), 3.22 (s, 3H), 2.78 (s, 3H).

^{13}C NMR (101 MHz, D2O) δ 163.78, 154.16, 141.78, 95.90, 47.26, 45.98, 36.24, 33.39.

LCMS-ESI m/z calculated for $C_9H_{15}N_5OH^+$ [M+H$^+$] 182.13, found 182.2.

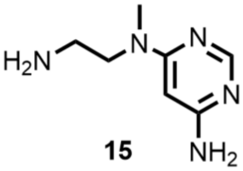

15

N4-(2-aminoethyl)-N4-methylpyrimidine-4,6-diamine dihydrochloride (15 2HCl): This compound was synthesized using general procedures **A** and **C**, with yields of 48 and 84% respectively.

^{1}H NMR (400 MHz, Deuterium Oxide) δ 8.22 (d, J = 0.8 Hz, 1H), 5.82 (d, J = 0.8 Hz, 1H), 4.03 (t, J = 5.9 Hz, 2H), 3.32 (t, J = 5.9 Hz, 2H), 3.13 (s, 3H).

^{13}C NMR (101 MHz, Deuterium Oxide) δ 162.03, 154.18, 148.13, 81.05, 46.98, 37.82, 36.22.

LCMS-ESI m/z calculated for $C_7H_{13}N_5H^+$ [M+H$^+$] 168.12, found 168.2.

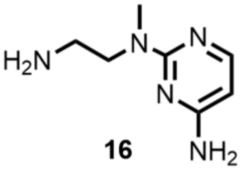

16

N2-(2-aminoethyl)-N2-methylpyrimidine-2,4-diamine dihydrochloride (16 2HCl): This compound was synthesized using general procedures **A** and **C**, with yields of 87 and 84% respectively.

^{1}H NMR (400 MHz, Deuterium Oxide) δ 7.67 (d, J = 7.3 Hz, 1H), 6.24 (d, J = 7.2 Hz, 1H), 4.01 (t, J = 5.7 Hz, 2H), 3.35 (t, J = 5.8 Hz, 2H), 3.20 (s, 3H).

^{13}C NMR (101 MHz, Deuterium Oxide) δ 164.49, 154.17, 141.54, 97.88, 47.27, 37.83, 35.08.

LCMS-ESI m/z calculated for $C_7H_{13}N_5$ [M+H$^+$] 168.12, found 168.2.

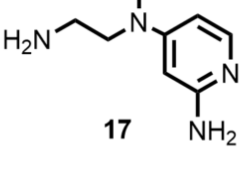

17

N4-(2-aminoethyl)-N4-methylpyridine-2,4-diamine dihydrochloride (17 2HCl): This compound was synthesized from the related 2-nitropyridine using general procedures **A**, **C**, and **D,** with yields of 75, 93, and 96% respectively.

^{1}H NMR (400 MHz, Deuterium Oxide) δ 7.58 (d, J = 7.6 Hz, 1H), 6.49 (dd, J = 7.6, 2.6 Hz, 1H), 5.98 (d, J = 2.6 Hz, 1H), 3.85 (t, J = 6.7 Hz, 2H), 3.32 (t, J = 6.7 Hz, 2H), 3.16 (s, 3H).

^{13}C NMR (101 MHz, Deuterium Oxide) δ 157.79, 153.14, 135.24, 100.72, 88.37, 48.38, 38.00, 36.52.

LCMS-ESI m/z calculated for $C_8H_{14}N_4H^+$ [M+H$^+$] 167.13, found 167.1.

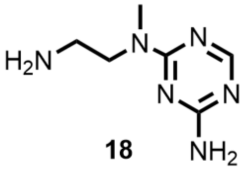

18

N2-(2-aminoethyl)-N2-methyl-1,3,5-triazine-2,4-diamine (18 2HCl): This compound was synthesized using general procedures **A** and **C**, with yields of 77 and 91% respectively

^{1}H NMR (400 MHz, Deuterium Oxide) δ 8.38 (d, J = 9.4 Hz, 1H), 4.10 (t, J = 5.9 Hz, 1H), 4.04 (t, J = 5.9 Hz, 1H), 3.39 (m, 2H), 3.32 (s, 1.5 H), 3.27 (s, 1.5 H).

^{13}C NMR (101 MHz, Deuterium Oxide) δ 162.76, 161.84, 156.07, 155.86, 155.42, 155.38 47.02, 37.54, 37.31, 35.64, 35.36. All peaks visibly doubled except 47.02.

LCMS-ESI m/z calculated for $C_6H_{12}N_6H^+$ [M+H$^+$] 169.12, found 169.0.

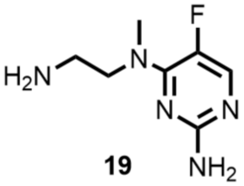

19

N4-(2-aminoethyl)-5-fluoro-N4-methylpyrimidine-2,4-diamine dihydrochloride (19 2HCl): This compound was synthesized using general procedures **A** and **C**, with yields of 94 and 84% respectively.

^{1}H NMR (400 MHz, Deuterium Oxide) δ 7.82 (d, J = 7.9 Hz, 1H), 4.06 (t, J = 6.1 Hz, 2H), 3.50 – 3.28 (m, 5H).

^{13}C NMR ^{13}C NMR (101 MHz, D_2O) δ 155.62 (m), 151.83 (s) 139.44 (d, *J* = 247.8 Hz), 128.07 (d, *J* = 38.4 Hz), 48.95 (d, *J* = 2.8 Hz), 37.77 (d, *J* = 12.3 Hz), 37.26 (s). (F-C aromatic splitting is analogous to Flucytosine)(4)

^{19}F NMR (376 MHz, D2O) δ -155.22.

LCMS-ESI m/z calculated for $C_7H_{12}FN_5H^+$ [M+H$^+$] 186.11, found 186.1.

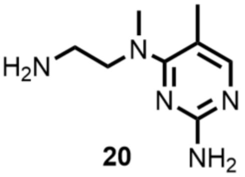

20

N4-(2-aminoethyl)-N4,5-dimethylpyrimidine-2,4-diamine dihydrochloride (20 2HCl): This compound was synthesized using general procedures **A** and **C**, with yields of 98 and 67% respectively.

^{1}H NMR (400 MHz, Deuterium Oxide) δ 7.50 (s, 1H), 4.02 (t, J = 5.8 Hz, 2H), 3.44 (s, 3H), 3.40 (t, J = 5.9 Hz, 2H), 2.33 (s, 3H).

^{13}C NMR (101 MHz, Deuterium Oxide) δ 165.01, 152.81, 141.50, 107.24, 49.54, 38.17, 37.38, 17.73.

LCMS-ESI m/z calculated for $C_8H_{15}N_5H^+$ [M+H$^+$] 182.14, found 182.2.

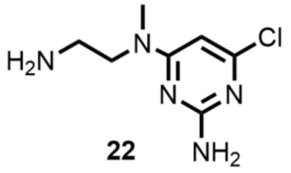

22

N4-(2-aminoethyl)-6-chloro-N4-methylpyrimidine-2,4-diamine (22 2HCl): This compound was synthesized using general procedures **A** and **C**, with yields of 97 and 44% respectively.

^{1}H NMR (400 MHz, Deuterium Oxide) δ 6.59 (s, 1H), 4.03 (t, J = 5.9 Hz, 2H), 3.36 (t, J = 6.0 Hz, 2H), 3.22 (s, 3H).

^{13}C NMR (101 MHz, Deuterium Oxide) δ 163.70, 155.08, 146.22, 95.34, 47.30, 37.53, 36.50.

LCMS-ESI m/z calculated for $C_7H_{12}ClN_5H^+$ [M+H$^+$] 202.09, found 202.2.

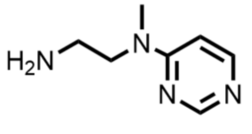

23

N1-methyl-N1-(pyrimidin-4-yl)ethane-1,2-diamine (23 2HCl): This compound was synthesized using general procedures **A** and **C**, with yields of 100 and 54% respectively.

^{1}H NMR (400 MHz, Deuterium Oxide) δ 8.73 (s, 1H), 8.23 (dd, *J* = 7.5, 1.6 Hz, 1H), 7.04 (d, *J* = 7.6 Hz, 1H), 4.22 (t, *J* = 6.0 Hz, 2H), 3.42 (t, *J* = 6.1 Hz, 2H), 3.34 (s, 3H).

^{13}C NMR (101 MHz, Deuterium Oxide) δ 163.00, 150.24, 142.36, 103.87, 47.52, 37.33, 36.74.

LCMS-ESI m/z calculated for $C_7H_{12}N_4H^+$ [M+H$^+$] 153.11, found 153.2.

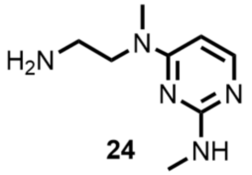

24

N4-(2-aminoethyl)-N2,N4-dimethylpyrimidine-2,4-diamine (24 2HCl): This compound was synthesized using general procedures **A** and **C**, with yields of 88 and 81% respectively.

^{1}H NMR (400 MHz, Deuterium Oxide) δ 7.71 (d, *J* = 7.6 Hz, 1H), 6.36 (d, *J* = 7.7 Hz, 1H), 4.16 – 3.90 (m, 2H), 3.39 (t, *J* = 6.0 Hz, 2H), 3.24 (s, 3H), 2.99 (s, 3H).

^{13}C NMR (101 MHz, Deuterium Oxide) δ 163.35, 153.39, 141.68, 94.89, 46.92, 37.54, 36.48, 27.23.

LCMS-ESI m/z calculated for $C_8H_{15}N_5$ [M+H$^+$] 182.14, found 182.2

Synthesis of 8

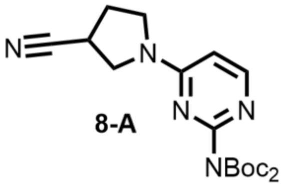

8-A

tert-butyl (tert-butoxycarbonyl)(4-(3-cyanopyrrolidin-1-yl)pyrimidin-2-yl)carbamate (8-A): This compound was synthesized using general procedure **A**, using tert-butyl (tert-butoxycarbonyl)(4-chloropyrimidin-2-yl)carbamate and pyrrolidine-3-carbonitrile TFA as starting material. The crude reaction mixture was purified by flash chromatography (silica gel, 12-100% EtOAc/Hexanes) to provide tert-butyl (tert-butoxycarbonyl)(4-(3-cyanopyrrolidin-1-yl)pyrimidin-2-yl)carbamate (295 mg, 0.76 mmol, 51%) as a white foam.

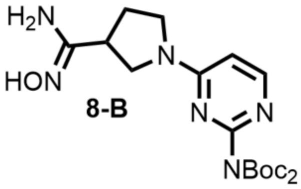

8-B

tert-butyl (4-(3-(N'-hydroxycarbamimidoyl)pyrrolidin-1-yl)pyrimidin-2-yl)carbamate (8-B): Tert-butyl (tert-butoxycarbonyl)(4-(3-cyanopyrrolidin-1-yl)pyrimidin-2-yl)carbamate (1.0 equiv., 141 mg, 0.36 mmol) was added to a pressure flask with EtOH (2.2 ml) and treated with hydroxylamine (4.69 equiv., 112 mg, 100 µL, 1.7 mmol). The reaction was stirred at 73°C for 15 hours at which time HPLC showed full conversion. The mixture was concentrated to dryness to afford a mixture of tert-butyl (4-(3-(N'-hydroxycarbamimidoyl)pyrrolidin-1-yl)pyrimidin-2-yl)carbamate (99 mg, 0.308 mmol, 85% estimated) and tert-butyl (tert-butoxycarbonyl)(4-(3-(N'-hydroxycarbamimidoyl)pyrrolidin-1-yl)pyrimidin-2-yl)carbamate (21 mg, 0.049 mmol, 13% estimated). This material was used in the subsequent step without purification.

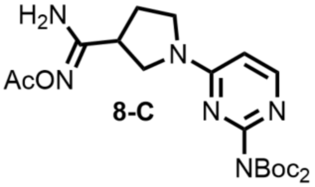

8-C

tert-butyl (4-(3-(N'-acetoxycarbamimidoyl)pyrrolidin-1-yl)pyrimidin-2-yl)carbamate (8-C): A cold (0°C) solution of tert-butyl (4-(3-(N'-hydroxycarbamimidoyl)pyrrolidin-1-yl)pyrimidin-2-yl)carbamate and its Bisboc analog (1.0 equiv., 0.357 mmol) in THF (2.0 ml) was treated with pyridine (1.28 equiv., 36 mg, 37 µL, 0.36 mmol) and acetic anhydride (1.19 equiv., 44 mg, 40 µL, 0.43 mmol). The reaction was warmed to room temperature and stirred for one hour. The solvent was removed *in vacuo* and the residue was purified by flash chromatography (silica gel, 1-10% MeOH/DCM) to provide to provide tert-butyl (4-(3-(N'-acetoxycarbamimidoyl)pyrrolidin-1-yl)pyrimidin-2-yl)carbamate (62 mg, 0.17 mmol, 47.7%) and tert-butyl (4-(3-(N'-

acetoxycarbamimidoyl)pyrrolidin-1-yl)pyrimidin-2-yl)(tert-butoxycarbonyl)carbamate (21 mg, 0.045 mmol, 12.7%). These products were combined in the next step.

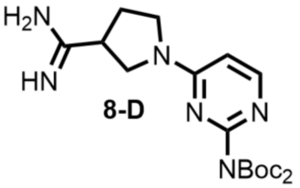

tert-butyl (4-(3-carbamimidoylpyrrolidin-1-yl)pyrimidin-2-yl)carbamate (8-D): Pd/C (0.065 equiv., 14 mg, 0.013 mmol) was added to a nitrogen purged solution of tert-butyl (4-(3-(N'-acetoxycarbamimidoyl)pyrrolidin-1-yl)pyrimidin-2-yl)carbamate and its Bisboc analog (1.0 equiv., 0.203 mmol) in EtOAc (2.5 ml). The flask was evacuated, backfilled with H_2 (3x), and stirred at room temperature under an H_2 balloon for 17 hours. After that time the reaction was mostly incomplete. The reaction was transferred to a Parr flask and the solution hydrogenated at 35-50 psi for 4 hours. After that time complete conversion of starting materials was observed. The excess hydrogen was evacuated from the flask (3x), the reaction filtered through a Celite ® pad, and the pad rinsed with EtOH. The solvent was removed in vacuo to furnish a mixture of tert-butyl (4-(3-carbamimidoylpyrrolidin-1-yl)pyrimidin-2-yl)carbamate (42 mg, 0.38 mmol, 68%) and tert- butyl (tert-butoxycarbonyl)(4-(3-carbamimidoylpyrrolidin-1-yl)pyrimidin-2-yl)carbamate (12 mg, 0.0284 mmol, 14%). This material was used in the subsequent step without purification.

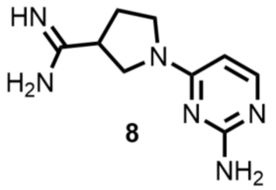

1-(2-aminopyrimidin-4-yl)pyrrolidine-3-carboximidamide (8 2HCl): This compound was synthesized using general procedure **C**. Crude product was purified on a Teledyne Isco Combiflash RF200 using the following conditions. The yield was 52%.

HPLC conditions:

Column: RediSep Column: C18 15.5g Gold

Mobile Phase: A – water B – ACN

Flow rate: 3.0 mL/minute

Gradient: 0-70% B from 0-10 minutes

^{1}H NMR (400 MHz, Deuterium Oxide) δ 7.71 (d, *J* = 7.3 Hz, 1H), 6.45 – 6.20 (m, 1H), 4.31 – 4.05 (m, 1H), 3.75 – 3.48 (m, 3H), 3.25 – 3.01 (m, 1H), 2.65 – 2.51 (m, 1H), 2.40 – 2.22 (m, 1H).

^{13}C NMR (101 MHz, Deuterium Oxide) δ 170.18, 169.98, 160.46, 160.38, 154.70, 141.00, 140.94, 97.06, 96.84, 49.35, 49.29, 46.56, 40.93, 40.21, 28.90, 28.31. All peaks visibly doubled except 154.70 and 46.56.

^{13}C NMR (free base) (101 MHz, Deuterium Oxide) δ 170.12, 160.44, 154.70, 141.45, 96.76, 49.26, 46.49, 40.20, 28.79.

LCMS-ESI m/z calculated for $C_9H_{14}N_6$ [M+H$^+$] 207.14, found 207.0

Synthesis of 21

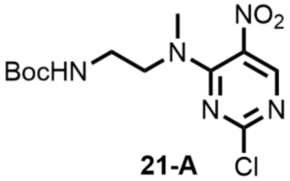

21-A

tert-butyl (2-((2-chloro-5-nitropyrimidin-4-yl)(methyl)amino)ethyl)carbamate (21-A): 2,4-dichloro-5-nitropyrimidine (1.0 equiv., 582 mg, 3.0 mmol) was dissolved in anhydrous DCM (10 ml) and cooled to -78°C. DIPEA (1.83 equiv., 710 mg, 0.96 ml, 5.49 mmol) and tert-butyl (2-(methylamino)ethyl)carbamate (1.0 equiv., 523 mg, 3.0 mmol) were dissolved in anhydrous DCM (10 ml) and added via addition funnel to the previous solution over 20 minutes. The reaction was stirred at -78°C for one hour before warming to 0°C over two hours while stirring. The reaction was poured into a separatory funnel and extracted with water (3x 10 ml) and brine (1x 20 ml). The organic layer was dried over Na_2SO_4, filtered, and the solvent removed to furnish tert-butyl N-{2-[(2-chloro-5- nitropyrimidin-4-yl)(methyl)amino]ethyl}carbamate (981 mg, 2.96 mmol, 99%) as a dark orange oil. This material was used in the subsequent step without purification.

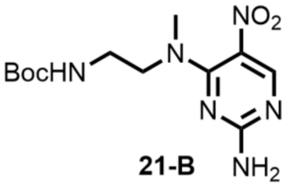

21-B

tert-butyl (2-((2-amino-5-nitropyrimidin-4-yl)(methyl)amino)ethyl)carbamate (21-B): tert-butyl N-{2-[(2-chloro-5- nitropyrimidin-4-yl)(methyl)amino]ethyl}carbamate (1. 0 equiv., 981 mg, 2.96 mmol) was dissolved in 40 ml of THF and 3 ml of 50% NH_4OH $_{(aq.)}$ solution was added. The reaction was stirred at room temperature for one hour before heating to 50°C and stirring an additional two hours. After this time 2 ml of 50% NH_4OH $_{(aq.)}$ solution was added and the reaction was stirred a further 2 hours. The reaction was cooled, the solvent removed *in vacuo*, and the crude residue purified by flash chromatography (2-7 % MeOH/DCM). Tert-butyl (2-((2-amino-5-nitropyrimidin-4-yl)(methyl)amino)ethyl)carbamate (528 mg, 1.69 mmol, 56%) was isolated as a yellow foamy gel.

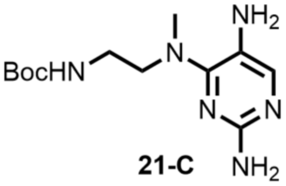

21-C

tert-butyl (2-((2,5-diaminopyrimidin-4-yl)(methyl)amino)ethyl)carbamate (21-C): This compound was synthesized using general procedure **D** on the 0.24 mmol scale. The crude residue was purified by flash chromatography (2-20 % MeOH/DCM). Tert-butyl (2-((2,5-diaminopyrimidin-4-yl)(methyl)amino)ethyl)carbamate (70 mg, 0.183 mmol, 75%) was isolated as a light pinkish tan foam.

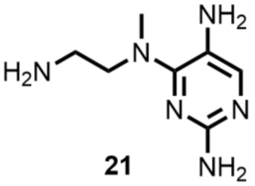

21

N4-(2-aminoethyl)-N4-methylpyrimidine-2,4,5-triamine dihydrochloride (21 2HCl): This compound was synthesized using general procedure **C** with a yield of 86%.

^{1}H NMR (400 MHz, Deuterium Oxide) ^{1}H NMR (400 MHz, D_2O) δ 7.65 (s, 1H), 4.05 (t, *J* = 5.9 Hz, 2H), 3.46 (s, 3H), 3.40 (t, *J* = 5.9 Hz, 2H).

^{13}C NMR (101 MHz, Deuterium Oxide) ^{13}C NMR (101 MHz, D_2O) δ 160.05, 151.86, 134.21, 134.18, 112.61, 49.61, 37.54, 37.02. ^{13}C NMR ~134.2 visibly doubled.

LCMS-ESI m/z calculated for $C_7H_{14}N_6H^+$ [M+H$^+$] 183.14, found 183.2.

Synthesis of pyrrolidine fragments of 1, 2, 3, and 4

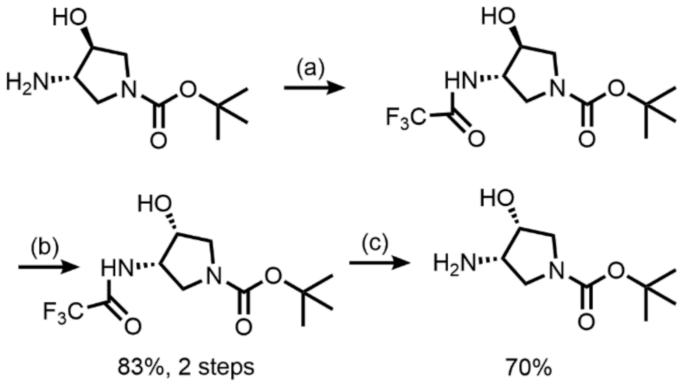

(a) TFAA, TEA, THF, 0-23°C, 16 h (b) MsCl, TEA, DBU, DCM, 0-23°C, 3 h (c) K_2CO_3, H_2O: MeOH, 23°C, 2.5 h.

Scheme S2: Synthesis of Tert-butyl (3S,4R)-3-amino-4-hydroxypyrrolidine-1-carboxylate.

Tert-butyl (3S,4R)-3-amino-4-hydroxypyrrolidine-1-carboxylate

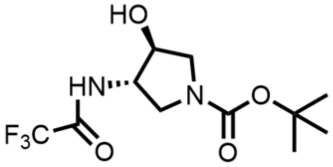

Step 1. A cold (0 °C) solution of tert-butyl (3S,4S)-3-amino-4-hydroxypyrrolidine-1-carboxylate (1 equiv., 3.55 g, 17.55 mmol) in THF (58 mL) was treated with TFAA (1.004 equiv., 3.7 g, 2.45 mL, 17.61 mmol) and TEA (1.209 equiv., 2.15 g, 2.95 mL, 21.22 mmol). The reaction warmed to room temperature, stirred overnight, and was monitored by TLC (20% MeOH/DCM). The reaction mixture was diluted with water (25 mL) and extracted with 10% IPA/$CHCl_3$ (140 mL, 2x). The combined organic layer was dried (Na_2SO_4) and concentrated *in vacuo*. The crude oil was dissolved in DCM and concentrated *in vacuo* (4x) to provide tert-butyl (3S,4S)-3-hydroxy-4-(2,2,2-trifluoroacetamido)pyrrolidine-1-carboxylate (5.24 g, 17.55 mmol).

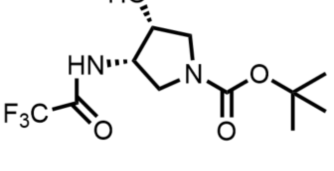

Step 2. A cold (0 °C) solution of crude tert-butyl (3S,4S)-3-hydroxy-4-(2,2,2-trifluoroacetamido)pyrrolidine-1-carboxylate (1 equiv., 5.23 g, 17.55 mmol) in DCM (120 mL) was treated with TEA (1.6 equiv., 2.84 g, 3.9 mL, 28.057 mmol) and MsCl (1.21 equiv., 2.44 g, 1.65 mL, 21.32 mmol). The reaction stirred for 5 min at this temperature and then for 2.5 hours at room temperature. DBU (3.013 equiv., 8.05 g, 7.9 mL, 52.88 mmol) was added, and stirring continued for 30 min at room temperature while the reaction was monitored by TLC (50% EtOAc/hexanes and 5% MeOH/DCM). The mixture was concentrated *in vacuo* and the remaining oil was diluted with minimal DCM and purified by flash chromatography (silica gel, 20-30% EtOAc/hexanes) to provide tert-butyl (3aS,6aR)-2-(trifluoromethyl)-3aH,4H,5H,6H,6aH-pyrrolo[3,4-d][1,3]oxazole-5-carboxylate (4.13 g, 14.74 mmol, 83% over 2 steps) as a pale yellow oil.

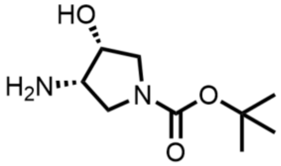

Step 3. A solution of tert-butyl (3aS,6aR)-2-(trifluoromethyl)-3aH,4H,5H,6H,6aH-pyrrolo[3,4-d][1,3]oxazole-5-carboxylate (1 equiv., 4.13 g, 14.74 mmol) in MeOH (55 mL) and water (27 mL) was treated with K_2CO_3 (6 equiv., 12.22 g, 88.42 mmol) and stirred at room temperature for 2.5 hours while monitoring by TLC (10% MeOH/DCM). The solvent was removed *in vacuo* and the resulting crude solid was diluted with 15% MeOH/DCM (10 mL), sonicated for 30 seconds, and the liquid was decanted off. This was repeated until TLC showed all the product was extracted from the solid (4x). The organic solution was concentrated *in vacuo* and the remaining oil was purified by flash chromatography (silica gel, 10-20% MeOH/DCM) to provide tert-butyl (3S,4R)-3-amino-4-hydroxypyrrolidine-1-carboxylate (2.07 g, 10.23 mmol, 70%) as an off-white foam.

^{1}H NMR (400 MHz, Methanol-*d*$_4$) δ 4.16 (td, J = 6.3, 2.0 Hz, 1H), 3.60 (dd, J = 10.4, 7.6 Hz, 1H), 3.56 – 3.39 (m, 2H), 3.36 (dd, J = 11.9, 4.2 Hz, 1H), 3.19 – 3.08 (m, 1H), 1.46 (s, 9H).

^{13}C NMR (400 MHz, Methanol-*d*$_4$) δ 154.96, 154.91, 79.70, 79.68, 70.04, 69.44, 53.13, 52.49, 52.47 52.11, 48.94, 48.42, 27.28. All peaks visibly doubled except 27.28, Rotomer.

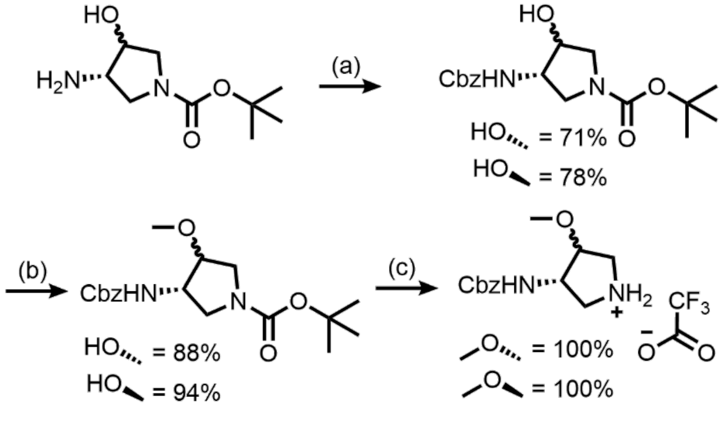

(a) CbzCl, Na_2CO_3, H_2O:Dioxane, 0-23°C, 3 h (b) Ag_2O, MeI, ACN, 23°C, 48 h (c) TFA: DCM, 23°C, 2 h.

Scheme S3: Synthesis of Pyrrolidine Fragments of 1, 2, 3, and 4.

Benzyl ((3S,4R)-4-methoxypyrrolidin-3-yl)carbamate

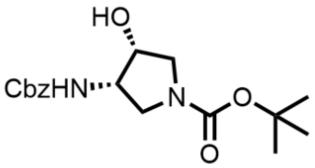

Step 1. To a cold (0 °C) solution of (3S,4R)-3-amino-4-hydroxypyrrolidine-1-carboxylate (1.0 equiv., 321 mg, 1.59 mmol) and sodium carbonate (1.2 equiv., 202 mg, 1.91 mmol) in water (3.65 mL) and dioxane (3.65 mL) was added benzyl chloroformate (1.2 equiv., 325 mg, 271 µL, 1.91 mmol) dropwise. The reaction was warmed to room temperature and stirred for 3 hours. The reaction was diluted with EtOAc (30 ml), the layers separated, and the aqueous layer back extracted with EtOAc (15 mL). The combined organic layers were washed with brine (15 mL), dried over anhydrous Na_2SO_4, filtered, and the solution concentrated *in vacuo*. The residue was purified by flash chromatography (silica gel, 12-100% EtOAc/Hexane) to provide tert-butyl (3S,4R)-3-(((benzyloxy)carbonyl)amino)-4-hydroxypyrrolidine-1-carboxylate (380 mg, 1.13 mmol, 71%) as a sticky white foam.

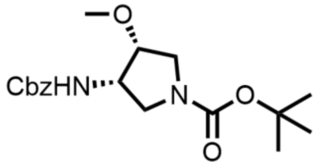

Step 2. Tert-butyl (3S,4R)-3-(((benzyloxy)carbonyl)amino)-4-hydroxypyrrolidine-1-carboxylate (1.0 equiv., 380 mg, 1.13 mmol) and Ag_2O (3.0 equiv., 785 mg, 3.39 mmol) were added to a foil covered flask with a stir bar. The flask was purged and backfilled with N_2 three times. Anhydrous ACN (10.9 ml) was added followed by MeI (10 equiv., 1604 mg, 703 µL 11.3 mmol). The reaction was stirred at room temperature for two days. The reaction was filtered through a pad of Celite ®, the pad rinsed with ACN (10 ml), and the solvent concentrated *in vacuo* using a rotary evaporator in a fume hood. The residue obtained was purified by flash chromatography (silica gel, 12-75% EtOAc/Hexane) to provide tert-butyl (3S,4R)-3-(((benzyloxy)carbonyl)amino)-4-methoxypyrrolidine-1-carboxylate (349 mg, 1.00 mmol, 88%) as a thick clear oil.

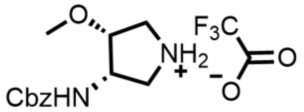

Step 3. To a stirring solution of tert-butyl (3S,4R)-3-(((benzyloxy)carbonyl)amino)-4-methoxypyrrolidine-1-carboxylate (1.0 equiv., 349 mg, 1.00 mmol) in DCM (2.4 ml) was added TFA (2.4 ml). The reaction was stirred at room temperature for two hours before the solvent was removed *in vacuo* directly. The residue was stripped with DCM (x4), MeOH (x2), and DCM (x2). The over-weight, light brown oil obtained was used directly is subsequent coupling steps assuming 100% yield.

Benzyl ((3S,4S)-4-methoxypyrrolidin-3-yl)carbamate

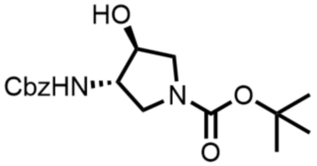

Step 1. To a cold (0 °C) solution of (3S,4S)-3-amino-4-hydroxypyrrolidine-1-carboxylate (1.0 equiv., 1100 mg, 5.44 mmol) and sodium carbonate (1.2 equiv., 692 mg, 6.53 mmol) in water (12.5 mL)

and dioxane (12.5 mL) was added benzyl chloroformate (1.2 equiv., 1113.3 mg, 930 µL, 6.53 mmol) dropwise. The reaction was warmed to room temperature and stirred for 3 hours. The reaction was diluted with EtOAc (100 ml), the layers separated, and the aqueous layer back extracted with EtOAc (60 mL). The combined organic layers were washed with brine (60 mL), dried over anhydrous Na_2SO_4, filtered, and the solution concentrated *in vacuo*. The residue was purified by flash chromatography (silica gel, 12-100% EtOAc/Hexane) to provide tert-butyl (3S,4S)-3-(((benzyloxy)carbonyl)amino)-4-hydroxypyrrolidine-1-carboxylate (1418 mg, 4.22 mmol, 78%) as a sticky white foam.

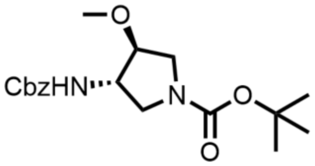

Step 2. Tert-butyl (3S,4S)-3-(((benzyloxy)carbonyl)amino)-4-hydroxypyrrolidine-1-carboxylate (1.0 equiv., 350 mg, 1.04 mmol) and Ag_2O (3.0 equiv., 723 mg, 3.12 mmol) were added to a foil covered flask with a stir bar. The flask was purged and backfilled with N_2 three times. Anhydrous ACN (10.0 ml) was added followed by MeI (10 equiv., 1476 mg, 650 µL 10.4 mmol). The reaction was stirred at room temperature for two days. The reaction was filtered through a pad of Celite ®, the pad rinsed with ACN (10 ml), and the solvent concentrated *in vacuo* using a rotary evaporator in a fume hood. The residue obtained was purified by flash chromatography (silica gel, 12-75% EtOAc/Hexane) to provide tert-butyl (3S,4S)-3-(((benzyloxy)carbonyl)amino)-4-methoxypyrrolidine-1-carboxylate (341 mg, 0.97 mmol, 94%) as a thick clear oil.

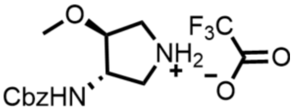

Step 3 To a stirring solution of tert-butyl (3S,4S)-3-(((benzyloxy)carbonyl)amino)-4-methoxypyrrolidine-1-carboxylate (1.0 equiv., 341 mg, 0.97 mmol) in DCM (2.4 ml) was added TFA (2.4 ml). The reaction was stirred at room temperature for two hours before the solvent was removed *in vacuo* directly. The residue was stripped with DCM (x4), MeOH (x2), and DCM (x2). The over-weight, light brown oil obtained was used directly is subsequent coupling steps assuming 100% yield.

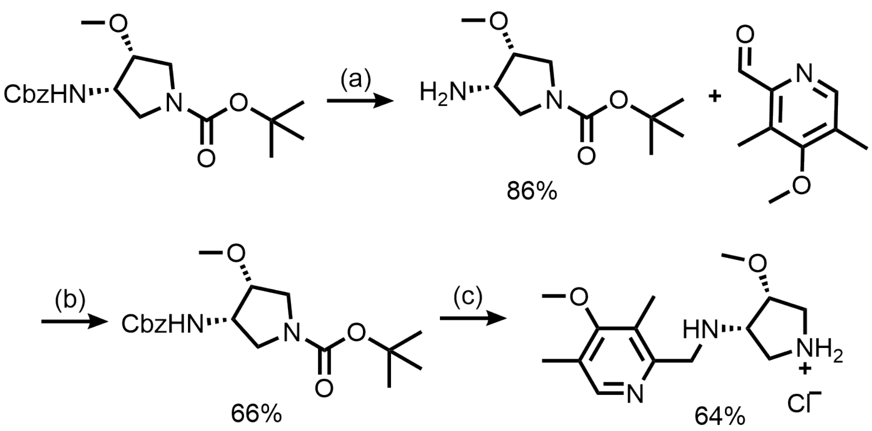

(a) $Pd(OH)_2/C$, Pd/C, H_2, EtOH, 40°C, 5 h. (b) STAB, AcOH, 4Å mol. seives, DCM, 23°C,16 h. (c) TFA:DCM, 0-23°C, 3 h: HCl.

Scheme S4: Synthesis of Fragment B.

(3S,4R)-4-methoxy-N-((4-methoxy-3,5-dimethylpyridin-2-yl)methyl)pyrrolidin-3-amine (B)

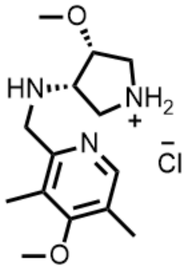

Step 1. A solution of (3S,4R)-3-(((benzyloxy)carbonyl)amino)-4-methoxypyrrolidine-1-carboxylate (1.0 equiv., 439 mg, 1.25 mmol) in EtOH (4 ml) was purged and backfilled with N_2 three times. Pd/C (0.15 equiv., 199 mg, 0.1875 mmol) and $Pd(OH)_2/C$ (0.05 equiv., 44 mg, 0.0625 mmol) were added. The reaction was purged and backfilled with H_2 three times using a balloon and T-valve. The reaction was stirred at 40 °C for 5 hours. The reaction as cooled to room temperature, purged, and backfilled with N_2 three times. The reaction was filtered through a pad of Celite ®, the pad rinsed with MeOH (10 ml), and the solvent concentrated *in vacuo. T*ert-butyl (3S,4R)-3-amino-4-methoxypyrrolidine-1-carboxylate (233 mg, 1.07 mmol, 86%) was obtained as a violet-gray oil and used without purification.

Step 2. To a solution of *t*ert-butyl (3S,4R)-3-amino-4-methoxypyrrolidine-1-carboxy (1.0 equiv., 162 mg, 0.75 mmol) and 4-methoxy-3,5-dimethylpicolinaldehyde (1.1 equiv., 135 mg, 0.825 mmol) in DCM (1.5 ml) was added ~200 mg of activated 3 Å molecular sieves. A drop of acetic acid was added, and the mixture was stirred at room temperature for 1 hour before the addition of sodium triacetoxyborohydride (1.5 equiv., 237 mg, 1.12 mmol). The reaction was stirred for 16 hours before dilution with DCM (10 ml), followed by washings with $NaHCO_3$ (1x 5ml) and brine (1x 5 ml). The organic layer was dried over anhydrous $MgSO_4$, filtered, the solution concentrated *in vacuo*. The was purified by flash chromatography (silica gel, 0-15% MeOH/EtOAc: 0-10% MeOH/DCM) to provide tert-butyl (3R,4S)-3-methoxy-4-(((4-methoxy-3,5-dimethylpyridin-2-yl)methyl)amino)pyrrolidine-1-carboxylate (181 mg, 0.50 mmol, 66%).

Step 3. To a cold (0 °C), stirring solution of tert-butyl (3R,4S)-3-methoxy-4-(((4-methoxy-3,5-dimethylpyridin-2-yl)methyl)amino)pyrrolidine-1-carboxylate (1.0 equiv., 39.6 mg, 0.108 mmol) in DCM (0.5 ml) was added TFA (0.5 ml) dropwise. The reaction was warmed to room temperature

and stirred for 3 hours. The solvent was concentrated *in vacuo*, stripped with DCM (x3), MeOH (x2) and 4 M HCl in dioxane (x2, ~0.3 ml). The residue was dissolved in water (3 ml), extracted with ether (x3, 1 ml), and the aqueous layer frozen and lyophilized. (3S,4R)-4-methoxy-N-((4-methoxy-3,5-dimethylpyridin-2-yl)methyl)pyrrolidin-3-amine trihydrochloride (25.7 mg, 0.069 mmol, 64%) was obtained as a light green foam.

^{1}H NMR (400 MHz, Methanol-*d*$_4$) δ: ^{1}H NMR (400 MHz, Methanol-*d*$_4$) δ 8.55 (s, 1H), 4.64 – 4.48 (m, 2H), 4.49-4.42 (m, 1H), 4.29-4.18 (m, 1H), 4.11 (s, 3H), 3.90 – 3.76 (m, 2H), 3.54 (s, 3H), 3.50 – 3.46 (m, 1H), 3.41-3.34 (m, 1H), 2.47 (s, 3H), 2.44 (s, 3H).

^{13}C NMR (101 MHz, Methanol-*d*$_4$) δ 170.37, 145.25, 143.05, 129.76, 129.26, 75.99, 66.71, 60.78, 58.51, 56.13, 45.32, 44.59, 13.18, 10.52.

HRMS-ESI m/z calculated for $C_{14}H_{23}N_3O_2$ H^+ [M+H$^+$] 266.18630, found 266.1859

Supplementary Figures

Figure S1

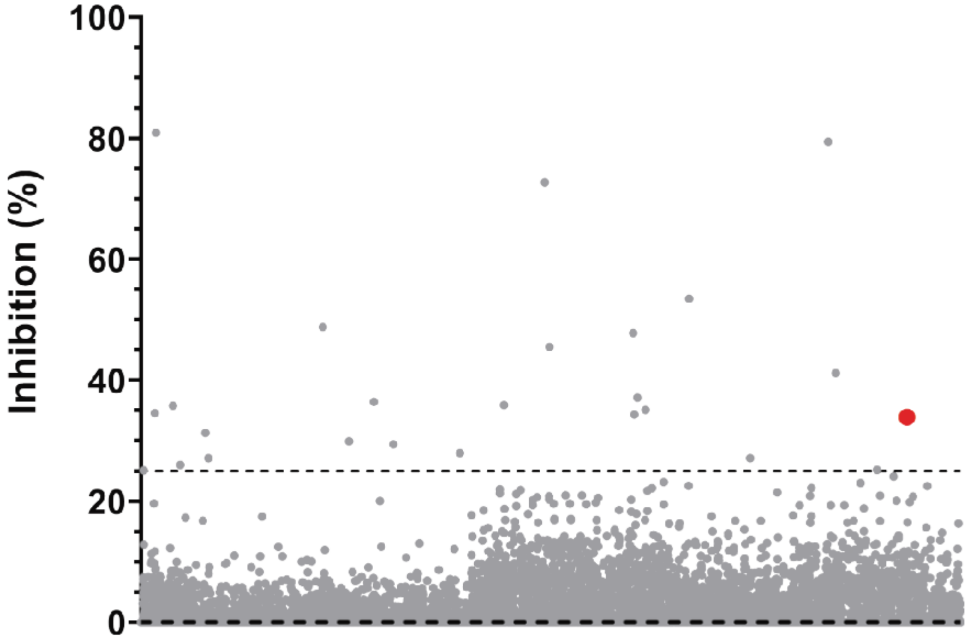

Fig. S1. Screening of Group I Intron Inhibitors. Scatter plot of high-throughput screening results of the 15K compounds in the Enamine RNA focused library. Hit threshold is shown as a dashed line. The red dot indicates the selected hit (Z3686288076) in this study.

Figure S2

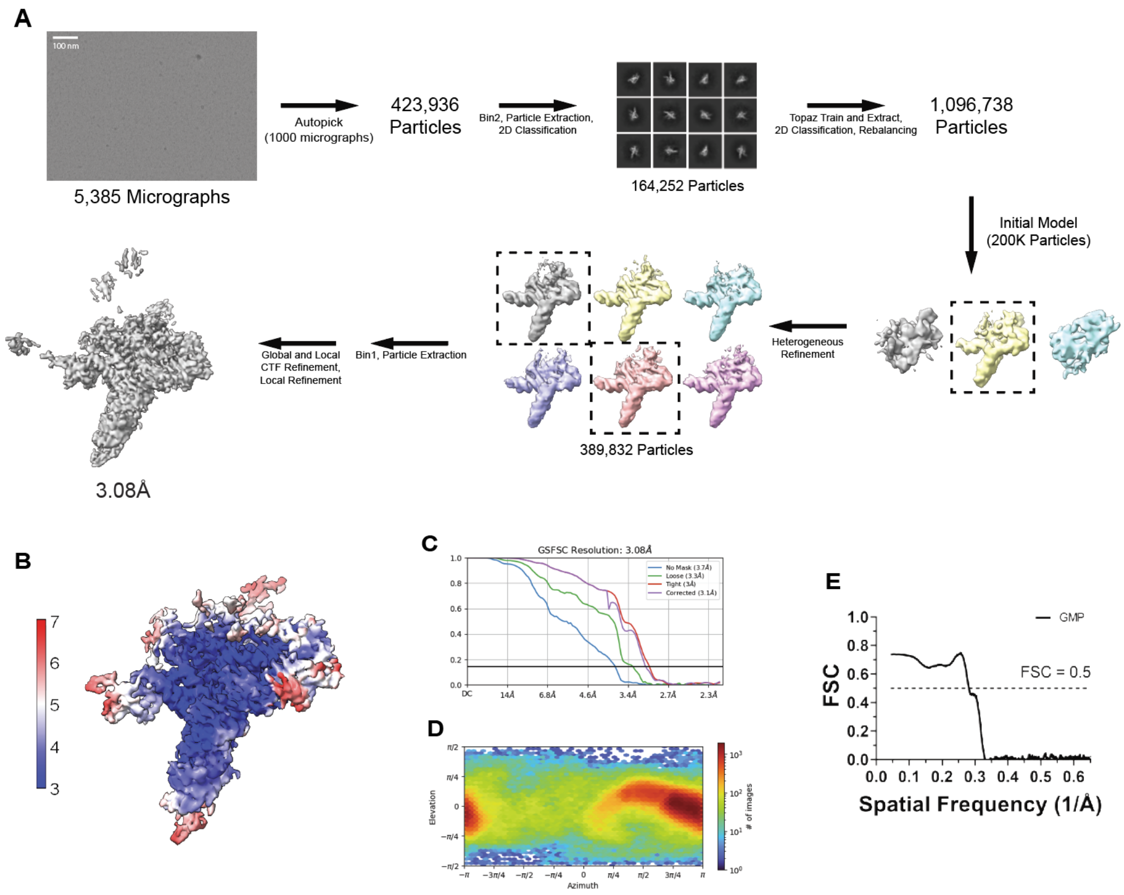

Fig. S2. CryoEM Workflow for the GMP-Group I Intron co-complex.
a. CryoEM data processing workflow for the group I intron bound to its natural ligand, GMP (see details in methods) **b.** Local resolution map of the GMP-group I intron co-complex. **c.** FSC curve for the cryoEM reconstruction of the GMP-intron co-complex. **d.** Particle orientation distribution of the GMP-intron structure. **e.** Map-to-model FSC curve for the GMP-intron structure.

Figure S3

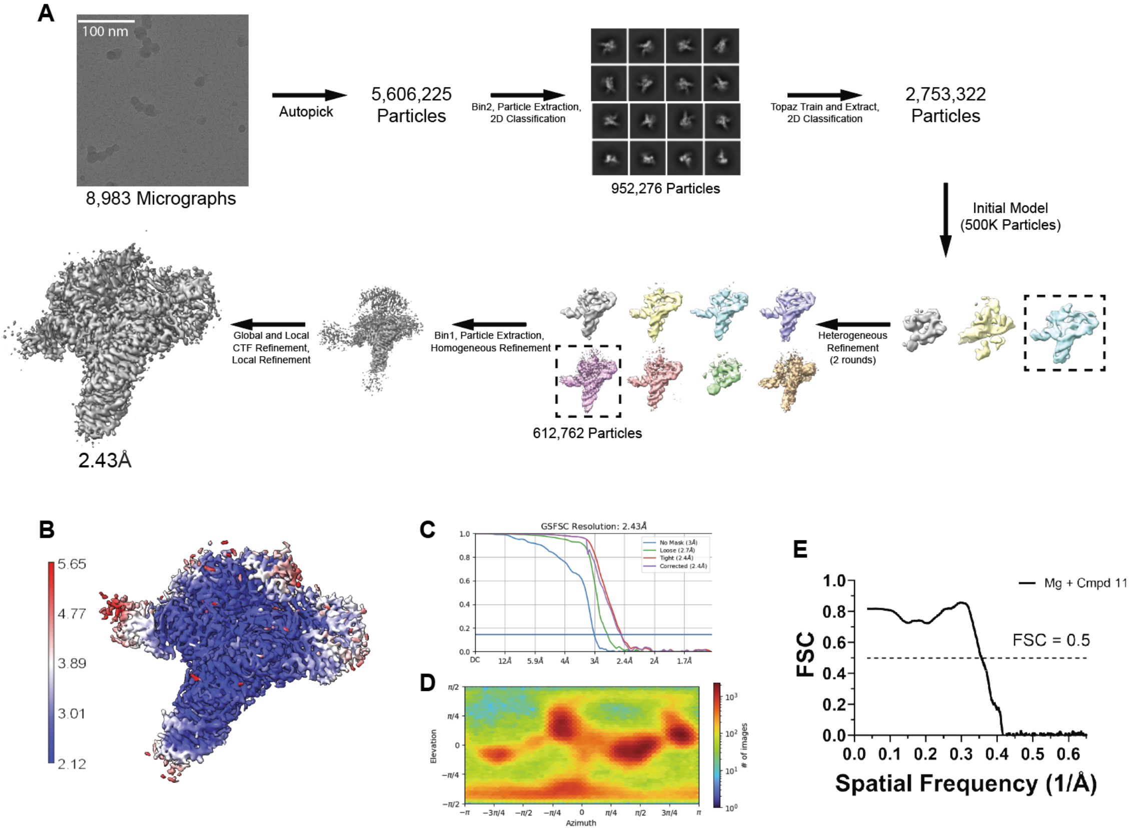

Fig. S3. CryoEM Workflow for the compound-11 Group I Intron co-complex (Mg).

a. CryoEM data processing workflow for the group I intron bound to compound **11** (see details in methods) **b.** Local resolution map of the compound **11**-group I intron co-complex. **c.** FSC curve for the cryoEM reconstruction of the compound **11**-intron co-complex. **d.** Particle orientation distribution of the compound **11**-intron structure. **e.** Map-to-model FSC curve for the compound **11**-intron structure.

Figure S4

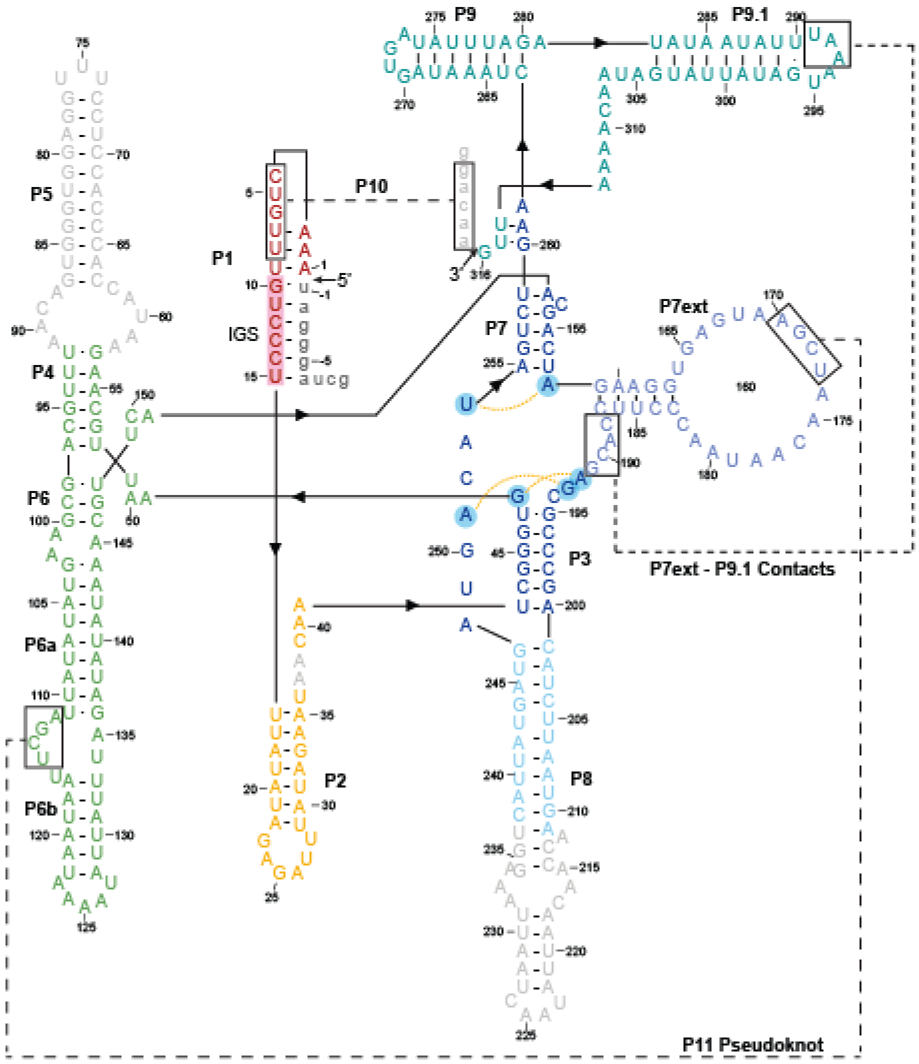

Fig. S4. Secondary Structure Diagram of the *C. albicans* Group I Intron.
The secondary structure diagram is prepared using the compound **11**-bound intron structure. Nucleotides are colored in the same color scheme as in Fig. 3. Splice sites are indicated by arrows. Internal guide sequence (IGS) within the intron is highlighted in pink shade. Unique tertiary interactions of the intron are indicated by boxed or shaded regions connected by dashed lines. Sequences displayed in light grey were not modeled into the compound 11-intron co-complex structure. 5′-exon sequences are in dark grey.

Figure S5

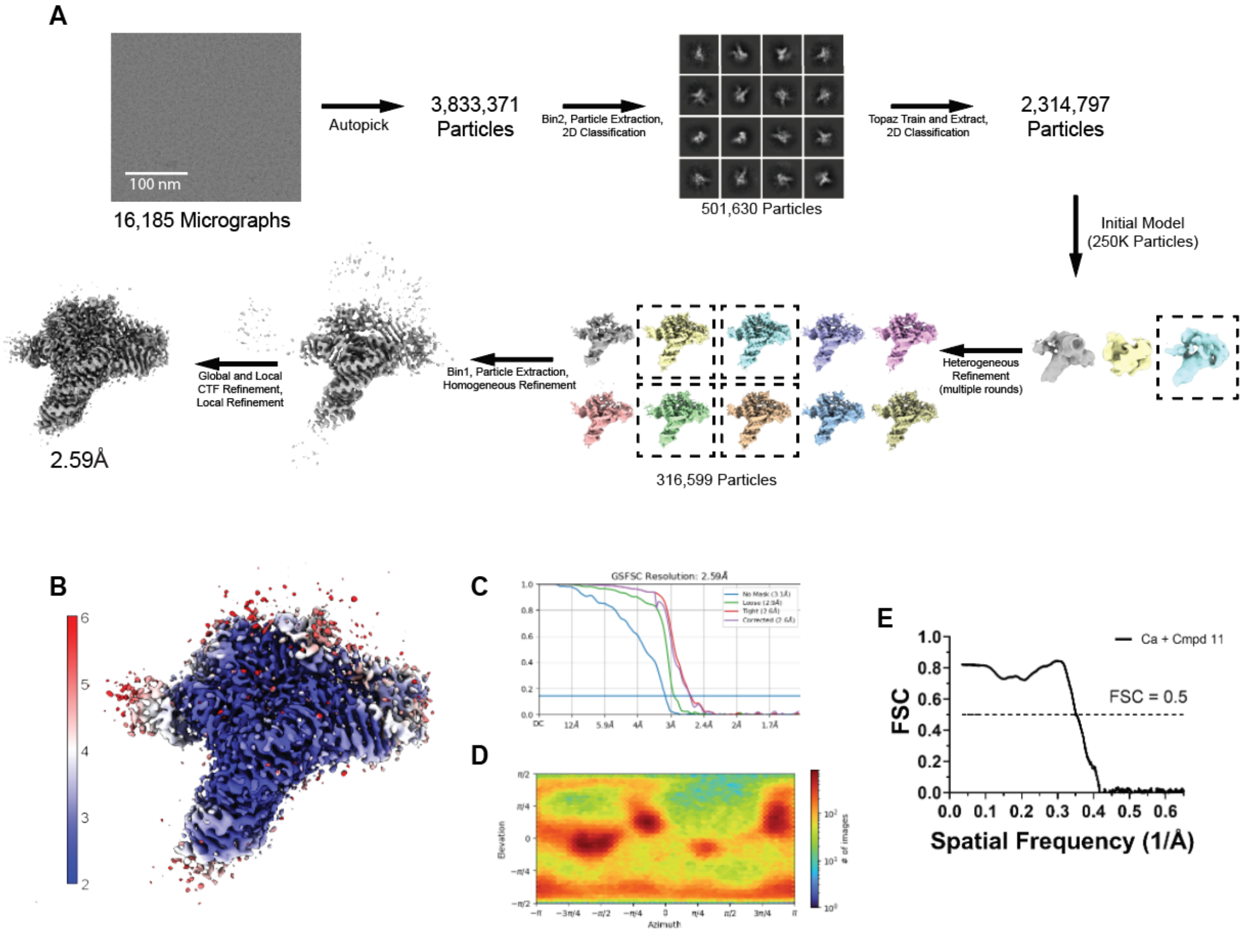

Fig. S5. CryoEM Workflow for the compound 11-Group I Intron co-complex (Ca).
a. CryoEM data processing workflow for the group I intron bound to compound **11** prepared with calcium in the buffer (see details in methods) **b.** Local resolution map of the compound **11** -group I intron co-complex. **c.** FSC curve for the cryoEM reconstruction of the compound **11** intron co-complex. **d.** Particle orientation distribution of the compound **11** intron structure. **e.** Map-to-model FSC curve for the compound **11** -intron structure.

Figure S6

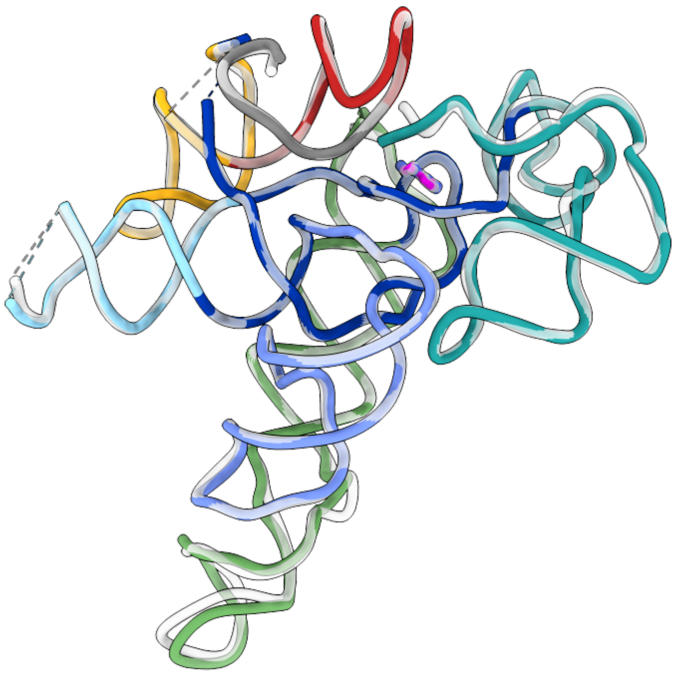

Fig. S6. Comparison of compound 11-bound structures in the presence of different divalent metal ions.

Comparison of the atomic models of the group I intron-compound **11** co-complex solved in magnesium (color) and calcium conditions (transparent).

Figure S7

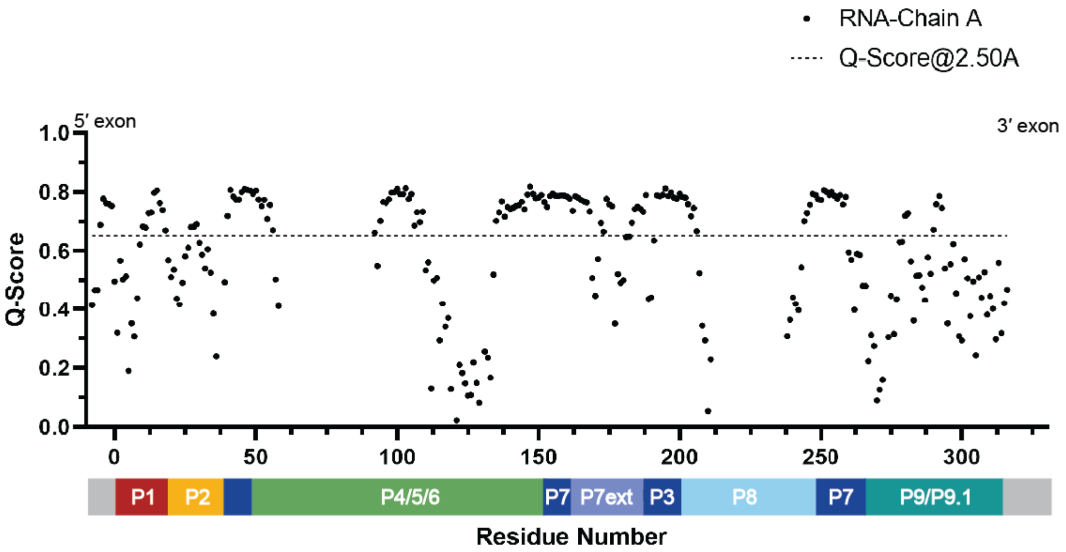

Fig. S7. Q-Score Analysis of the compound 11-Group I Intron Atomic Model (Mg).
Q-score analysis for the RNA chain of the compound **11**-group I intron co-complex. The domain organization of the intron is mapped in color to the corresponding residue number ranges. The expected Q-score value at a resolution of 2.5Å is plotted as a dashed line on the graph. Missing points on the graph correspond to regions of the intron that were not modelled.

Figure S8

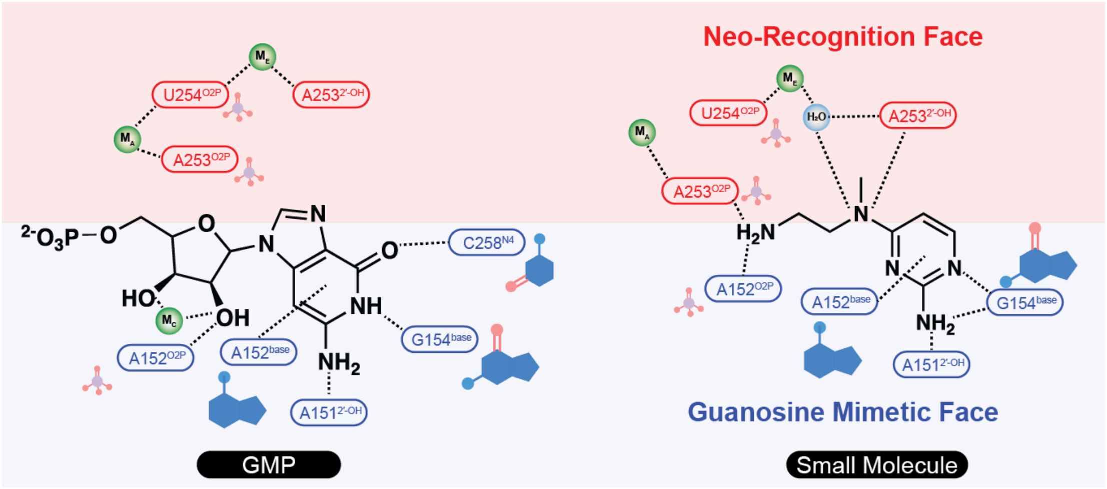

Fig. S8. Group I Intron Ligand Interaction Network.
Interactions of GMP (left) and compound **11** (right) with surrounding residues, metal ions and coordinated water molecules within the intron active site depicted.

Figure S9

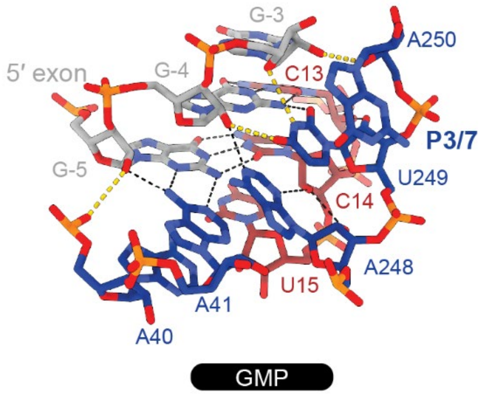

Fig. S9. P1 Docking Interactions in Different Liganded States.
Details of the interactions at the basal stem of P1 in the GMP structure. Yellow dashed lines indicate contacts that are broken in the compound **11**-bound structure.

Movie S1. Group I Intron in complex with compound 11 (Supplemental to Figures 4B and 4C). This movie presents the cryo-EM structure of the group I intron bound to compound **11**, featuring both the density map and molecular model. The group I intron and compound **11** are color-coded as in Figure 4A for consistency. A 360° rotation provides an overview of the co-complex structure, followed by a focused view of the compound **11** binding pocket. In this region, strong electron density (magenta mesh) clearly defines the precise positioning of the compound **11** within the pocket. Structural visualization was performed using UCSF ChimeraX, as mentioned in the Methods section.

Table S1. Small Molecule Screening Data

Category	Parameter	Description
Assay	Type of assay	*In vitro*
	Target	*Candida albicans* mitochondrial LSU.I1 group I intron
	Primary measurement	Detection of fluorescence upon self-splicing and subsequent molecular beacon hybridization to the ligated exon product
	Key reagents	*In vitro*-transcribed intron precursor RNA, chemically synthesized DNA oligo dual-labelled with 5' Alexa Fluor 555 and 3' Black Hole Quencher 2, reaction buffer (50 mM K-HEPES pH 7.5, 150 mM KCl, 3 mM $MgCl_2$ and 5% DMSO)
	Assay protocol	Small molecule stocks were dispensed to the assay plates (Corning 3575) using Echo 550 (Labcyte)
		Reaction buffer and precursor RNA stock (final concentration of 75 nM) were dispensed to the assay plates using Multidrop Combi (Thermo)
		Incubate at RT for 5 minutes to allow intron folding
		Guanosine stock was added to the final concentration of 20 µM to initiate the reaction and the plates were incubated at RT for 30 minutes
		EDTA stock was added to the final concentration of 5 mM to completely quench the reaction
		Molecular beacon stock was added to the final concentration of 75 nM (final liquid volume in each well is 20 µM)
		The plates were incubated at 75°C for 5 minutes and then returned to RT for 30 minutes to allow DNA:RNA hybrid formation
		Fluorescence intensity was read on a Synergy Neo2 plate reader with a customized optical filter (excitation: 540 nm, bandwidth: 5 nm; emission: 590 nm, bandwidth: 5 nm)
	Additional comments	Refer to Omran, Q. et al. Nucleic Acids Res. 2022 (PMID: 35438748) for considerations of setting up the molecular beacon assay
Library	Library size	15,520
	Library composition	RNA focused compounds
	Source	Enamine
	Additional comments	
Screen	Format	384-well plate (Corning 3575)
	Concentration(s) tested	20 µM compound, 5.2% final DMSO%
	Plate controls	Untreated wells (no compound) as negative control; No self-splicing precursor RNA (molecular beacon only) as positive control
	Reagent/ compound dispensing system	Multidrop Combi (Thermo) for reagent dispensing, Echo 550 (Labcyte) for compound dispensing
	Detection instrument and software	Synergy Neo2 (Biotek), Gen5 software (Biotek, version 3.02.1)
	Assay validation/QC	Z' score; average Z' score of the screen was 0.76
	Correction factors	Not applicable
	Normalization	Data are normalized relative to the negative control (0% inhibition) and to the positive control (100% inhibition)
	Additional comments	Screen was carried out at the Yale Center for Molecular Discovery (YCMD)
Post-HTS analysis	Hit criteria	3 standard deviations above the negative control
	Hit rate	0.2%
	Additional assay(s)	Radioanalytical self-splicing assay with dose titration of hit compounds
	Confirmation of hit purity and structure	Compounds were repurchased (Enamine, Chemspace)

Additional comments

Table S2. Biochemical *In Vitro* Evaluation of Hit and Compounds with Radioanalytic Splicing Assay.

Compound ID	Structure	IC50 (µM)	Does-dependent inhibition
Z3686288076	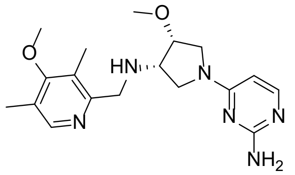	0.84	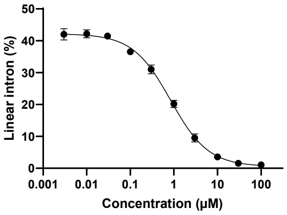
Fragment_RingC	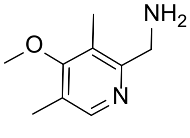	>100[a]	N/A
1	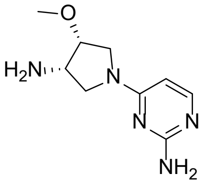	0.24	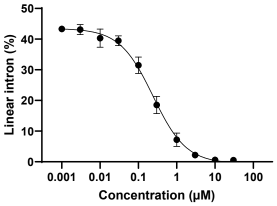
2	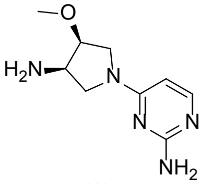	0.23	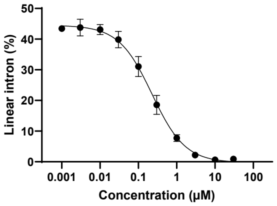
3	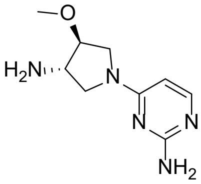	14.40	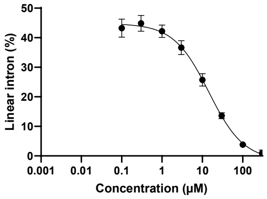
4	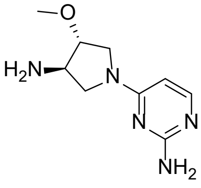	12.13	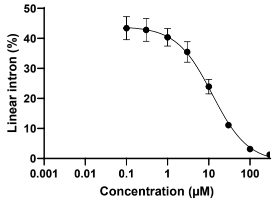

5	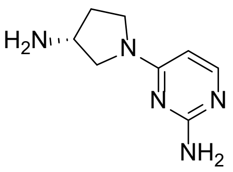	0.60	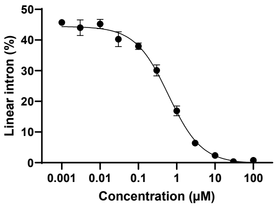
6	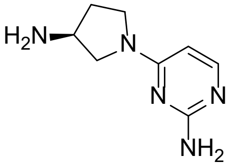	0.42	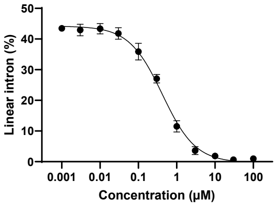
7	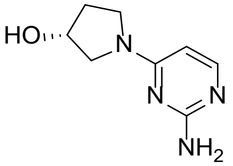	>100	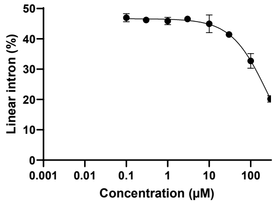
8	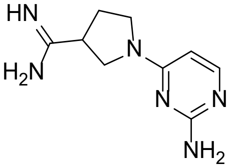	5.15	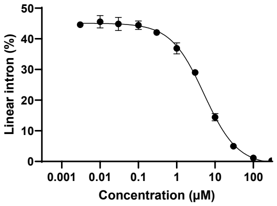
9	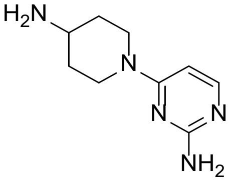	0.23	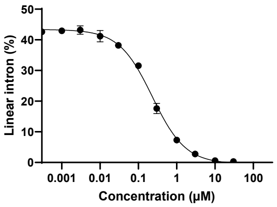
10	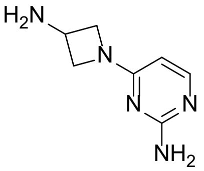	3.65	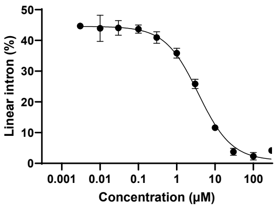
11	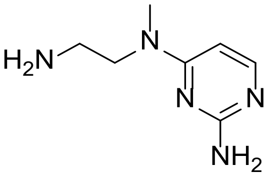	0.21	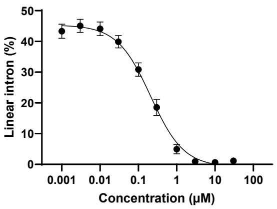

12	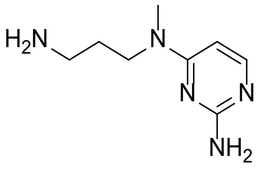	5.63	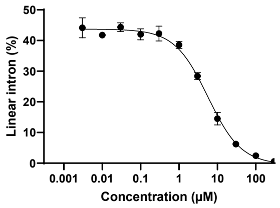
13	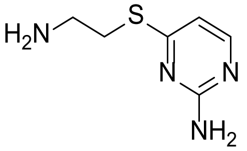	>100	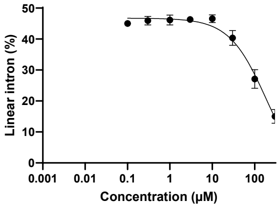
14	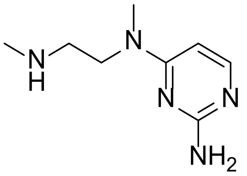	0.97	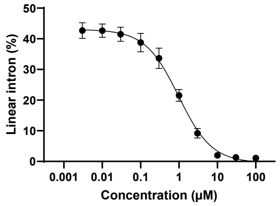
15	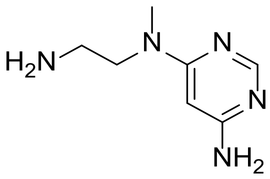	20.04	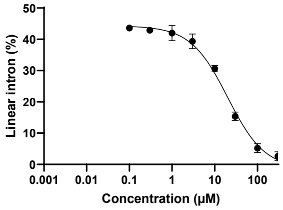
16	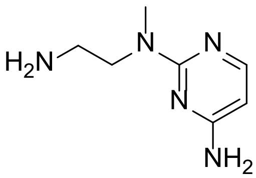	60.35	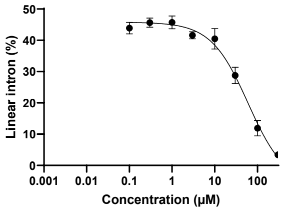
17	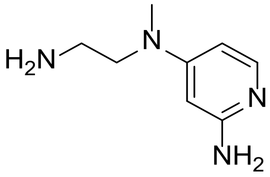	0.08	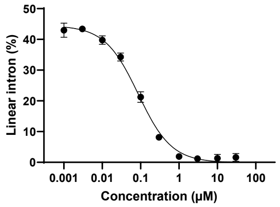
18	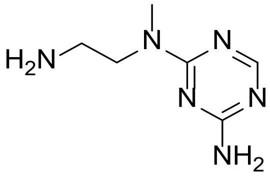	>100	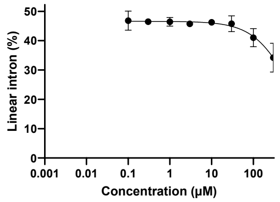

19	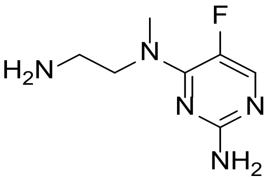	8.28	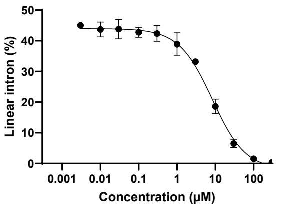
20	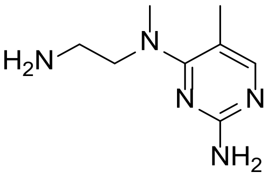	0.30	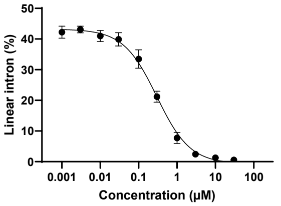
21	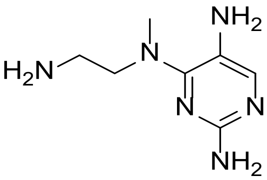	0.33	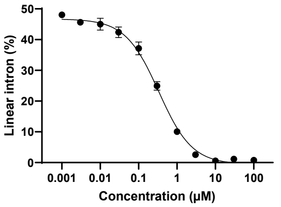
22	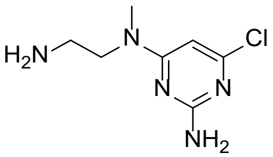	>100	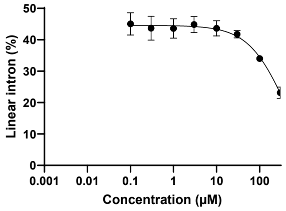
23	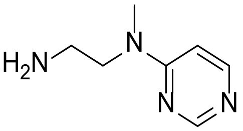	>100	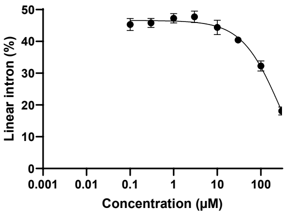
24	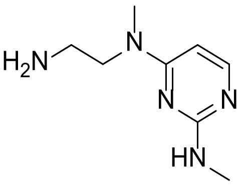	13.10	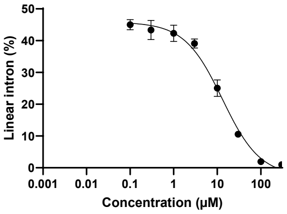

[a]IC50 was described as >100 μM when the IC50 cannot be determined within the range of concentrations tested.

Table S3. CryoEM Data Collection and Processing Parameters

	GMP-Intron Co-Complex	SM Intron Co-Complex (Magnesium)	SM Intron Co-Complex (Calcium)
**EMDB**	**EMD-48538**	**EMD-48539**	**EMD-48540**
**PDB**	**9MQS**	**9MQT**	**9MQU**
**Data Collection and Processing**			
**Microscope**	Titan Krios	Titan Krios	Titan Krios
**Voltage (kV)**	300	300	300
**Camera**	K3	Falcon 4i	Falcon 4i
**Magnification**	81,000	165,000	165,000
**Pixel Size (Å)**	1.068	0.743	0.743
**Total Electron Exposure (e-/A^2)**	59.3	50	50
**Number of Frames Collected**	43	40	40
**Defocus Range (µm)**	-1 to -2.5	-0.5 to -2	-0.5 to -2
**Symmetry Imposed**	C1	C1	C1
**Micrographs Collected**	5,385	8,983	16,185
**Micrographs Used**	5,239	8,569	7,844
**Initial Particles**	423,936	5,606,225	3,833,371
**Particles After 2D Classification**	1,096,738	2,753,322	2,314,797
**Particles for 3D Refinement**	389,832	612,762	316,599
**Refinement**			
**Map Resolution**	3.08	2.43	2.59
**FSC Threshold**	0.143	0.143	0.143
**Map sharpening B-factor (A^2)**	-99.3	-84.5	-87.6
**FSC Map to Model**	3.3	2.8	2.9
**FSC Threshold**	0.5	0.5	0.5
**Model Composition**			
**Non-hydrogen atoms**	5,201	5,664	5,658
**RNA bases**	244	264	264
**B factors (A^2)**			
**Nucleotide**	108.9	24.6	28.32
**Ligand**	57.84	7.18	13.93
**R.m.s. Deviation**			
**Bond length (Å)**	0.006	0.009	0.008
**Bond angles (°)**	0.972	1.152	1.119
**Validation**			
**Molprobity score**	2.53	2.45	2.46
**Clashscore**	6.91	5.55	5.67

References

1. U. von Ahsen, J. Davies, R. Schroeder, Non-competitive inhibition of group I intron RNA self-splicing by aminoglycoside antibiotics. *J Mol Biol* **226**, 935-941 (1992).
2. Q. Q. Omran, O. Fedorova, T. Liu, A. M. Pyle, A molecular beacon assay for monitoring RNA splicing. *Nucleic Acids Res* **50**, e74 (2022).
3. J. H. Zhang, T. D. Chung, K. R. Oldenburg, A Simple Statistical Parameter for Use in Evaluation and Validation of High Throughput Screening Assays. *J Biomol Screen* **4**, 67-73 (1999).
4. A. Harsanyi *et al.*, One-Step Continuous Flow Synthesis of Antifungal WHO Essential Medicine Flucytosine Using Fluorine. *Organic Process Research & Development* **21**, 273-276 (2017).
